# Effectiveness of SGLT2 inhibitors in slowing chronic kidney disease progression and reducing mortality: a meta-analysis

**DOI:** 10.3389/fphar.2026.1923548

**Published:** 2026-08-31

**Authors:** Arjun Sharma, Bharat Sharma, Deepthi Sree Vamaraju, Amrendra Kumar Ajay

**Affiliations:** 1 Harvard Medical School, Harvard University, Boston, MA, United States; 2 Division of Hemostasis and Thrombosis, Beth Israel Deaconess Medical Center, Boston, MA, United States; 3 Gori Devi Girls College, University of Rajasthan, Jaipur, Rajasthan, India; 4 Institute of Systems Science, National University of Singapore, Singapore, Singapore; 5 Department of Medicine, Brigham and Women’s Hospital, Boston, MA, United States

**Keywords:** chronic kidney disease (CKD), end-stage kidney disease (ESKD), estimated glomerular filtration rate (eGFR), renal progression, SGLT2 inhibitors

## Abstract

**Background:**

Chronic kidney disease (CKD) represents a significant health problem worldwide with high morbidity, mortality, and development of end-stage kidney disease (ESKD). Sodium-glucose co-transporter 2 (SGLT2) inhibitors represent an innovative class of medications with possible cardiorenal advantages. We conducted a Meta-analysis to assess their efficacy in CKD management.

**Methods:**

Our Meta-analysis was performed using the Preferred Reporting Items for Meta-analyses (PRISMA) guidelines and is registered in PROSPERO (CRD420261362785). Databases used for conducting this systematic review include Cochrane Central Register of Controlled Trials (CENTRAL), PubMed, PubMed Central, and Google Scholar. The databases were searched from their inception till 16 April 2026. Clinical trials in adults diagnosed with CKD were included in this study if they compared the use of SGLT2 inhibitors versus placebo. A random-effects model was used for meta-analysis.

**Results:**

Thirteen randomized controlled trials (RCTs) (n = 49,931) were analyzed. A statistically significant increase in total and chronic estimated glomerular filtration rate (eGFR) slope was seen (mean difference: MD = 0.97 mL/min/1.73 m^2^/year, 95% CI: 0.34 to 1.59 and MD = 1.92, 95% CI: 1.91–1.93). There was a statistically significant reduction in albuminuria (ratio of means showing 37% reduction, 95% CI: 1.12–1.68). The risk of ESKD was significantly reduced by 30% (RR = 0.70, 95% CI: 0.65–0.75), while the doubling of serum creatinine risk was diminished by 33% (RR = 0.67, 95% CI: 0.56–0.79). SGLT2 inhibitors also lowered the risk of all-cause mortality (RR = 0.80, 95% CI: 0.68–0.93), cardiovascular mortality (RR = 0.79, 95% CI: 0.65–0.95), and heart failure-related hospitalization (RR = 0.74, 95% CI: 0.67–0.82). The number of serious adverse events decreased (RR = 0.92, 95% CI: 0.88–0.95), while overall adverse events remained unchanged (RR = 0.99, 95% CI: 0.96–1.02). However, there was evidence of higher risk of developing diabetic ketoacidosis (RR = 2.06, 95% CI: 1.19–3.58) and volume depletion (RR = 1.28, 95% CI: 1.13–1.45).

**Conclusion:**

SGLT2 inhibitors were effective in delaying the progression of CKD and lowering mortality. However, despite having a relatively safe profile, this drug class comes with certain hazards that need to be monitored clinically.

**Systematic Review Registration:**

https://www.crd.york.ac.uk/prospero/, identifier CRD420261362785.

## Introduction

Chronic kidney disease (CKD) was reported in approximately 788 million adults worldwide in 2023, representing 14.7% of the global adult population aged ≥20 years of age, and indicating a substantial increase since 1990 (Mark et al., 2025). CKD is highly prevalent in North Africa and the Middle East where it is estimated to affect up to 18% of the population, mostly cases with early-stage CKD (stages 1–3) (Mark et al., 2025). Prevalence of CKD is about 14%–15% of the US adult population, but it is higher in patients having cardiometabolic risk factors, including hypertension and diabetes. However, awareness of CKD is relatively low in young adults, females, and minorities (Gong et al., 2026). Characterized by the gradual and irreversible loss of kidney function, CKD usually leads to electrolyte imbalance, anemia, bone mineral disorder, and finally end-stage kidney disease (ESKD) which necessitates dialysis or transplantation (Chen et al., 2019). Cardiovascular disease (CVD) is the predominant cause of mortality in patients with CKD due to conventional risk factors (hypertension, diabetes) as well as CKD-related risk factors (inflammation, uremia, bone mineral disorders, and volume overload) (Zoccali et al., 2023).

Treatment modalities for CKD involve regulation of blood pressure, lowering of proteinuria, and management of blood glucose in case of diabetes. Drugs like renin-angiotensin-aldosterone system (RAAS) inhibitors, which include angiotensin-converting enzyme (ACE) inhibitors and angiotensin II receptor blockers, have been the mainstay for years due to their nephroprotective properties. However, their application might be restricted in certain individuals owing to side effects, including hypotension, hyperkalemia, and acute kidney injury. These effects may require dose reduction or cessation of treatment, restricting their renal protective potential (Zhang et al., 2017). Furthermore, combined RAAS blockade does not offer any advantage in terms of clinical outcomes (Leoncini et al., 2020).

In recent times, sodium-glucose co-transporter 2 (SGLT2) inhibitors have gained prominence as a novel therapeutic approach with immense clinical utility beyond glucose regulation. Extensive randomized controlled trials (RCTs) indicate that these drugs minimize the chances of CKD advancement by approximately 45%, reduce hospital admissions due to heart failure by 23%, and diminish major cardiovascular events mostly in individuals with prior atherosclerotic cardiovascular disease (Zelniker et al., 2019). The possible preventive effects of SGLT2 inhibitors include decreasing glomerular hyperfiltration and glomerular pressure, increasing cortical oxygen supply, encouraging natriuresis and diuresis, reducing arterial pressure, and regulating inflammation and oxidation (Vallon and Verma, 2021). SGLT2 inhibitors induce metabolic alterations that favor ketone body utilization, thereby improving myocardial and renal energy metabolism and contributing to their cardiorenal protective effects (Salvatore et al., 2022). This effect is applicable to both diabetic and non-diabetic patients regardless of the kidney’s functional level, rendering SGLT2 inhibitors an essential treatment option for cardiorenal risk management (Caruso and Giorgino, 2022).

Even though there is extensive literature to support the efficacy and effectiveness of SGLT2 inhibitors, there is still a need for a thorough synthesis and evaluation of all available information, since clinical trials may differ in terms of methodology, target population, endpoints, and study duration. Moreover, despite the benefits of SGLT2 inhibitors on patients’ kidney health and cardiovascular outcomes being well established, further research is necessary when it comes to the safety profile and appropriate usage of the drug in practice (Kaze et al., 2022; Menne et al., 2019). To address this gap, the current systematic review and meta-analysis seek to assess whether SGLT2 inhibitors are effective in delaying the progression of CKD and lowering mortality among affected patients. Through meta-analysis of RCTs, this study aimed for an in-depth evaluation of the efficacy of SGLT2 inhibitors based on various clinical endpoints. The results of this study are anticipated to add to the existing scientific knowledge on the benefits of using SGLT2 inhibitors and help inform decisions by clinicians, policymakers, and researchers on how best to manage and treat CKD patients.

## Methods

### Search strategy and selection criteria

The systematic review was prospectively registered in PROSPERO (CRD420261362785) and followed the guidelines provided in the Preferred Reporting Items for Systematic Review and Meta-analyses (PRISMA) statement (Moher et al., 2010). An electronic database search was conducted utilizing three literature databases, namely, the Cochrane Central Register of Controlled Trials (CENTRAL), PubMed, and PubMed Central (Supplementary Appendix A). Additionally, Google Scholar was searched for potential references to ensure comprehensive literature retrieval and identification of citations that may have been missed during the bibliographic database search. Several Boolean search terms, including a combination of both MeSH and keywords depending on the respective literature database, regarding CKD, renal insufficiency, SGLT2 inhibitors, ESKD, and progression markers of CKD, like estimated glomerular filtration rate (eGFR) were used to optimize the search process. The search included all publications until 16 April 2026. Additional sources were also identified from reference searches in selected studies.

The abstracts of the articles were independently screened by two authors. Duplicates were removed, and data were independently extracted from the articles by the researchers. Any discrepancies were resolved by mutual consensus. The eligibility criteria for selecting the articles for this study were to include studies in English that had SGLT2 inhibitors as the main intervention, along with information about their effects on progression of CKD, renal parameters, and/or mortality rates. Articles that reported studies on adult populations, aged at least 18 years, with CKD were selected for analysis. As the RCTs included in this analysis were conducted in different time frames and various clinical settings, a standardized definition of CKD was not mandated in this review. Rather, the process of identifying and categorizing the stages of CKD was left to the criteria used within each specific study, which typically involved tests of kidney function like eGFR, albuminuria, or both. Reviews and commentary articles without primary data were not considered. Moreover, animal and *in vitro* experiment data and studies were excluded from this review (Supplementary Appendices B,C).

### Data extraction and analysis

The information was independently and manually extracted by two authors. The extracted terms included the first author of the paper, year of publication, location of the study, length of follow-up, and characteristics of the study population, including the number, age range or average age, and sex of participants, baseline eGFR (mL/min/1.73 m^2^), baseline urine albumin-to-creatinine ratio (UACR) (mg/g), and comorbidities. Information extracted from the interventions included SGLT2 inhibitor use and its dose and frequency of administration.

Clinical outcomes included total and chronic eGFR slope, relative change in UACR, incidence of ESKD (progression to dialysis or transplant), doubling of serum creatinine, heart failure-related hospitalization, all-cause death, cardiovascular death, adverse events, and serious adverse events. The specific side effect data comprised diabetic ketoacidosis (DKA), urinary tract infection (UTI), volume depletion, genital infection, bone fracture, amputation, and renal side effects. The Cochrane risk of bias assessment tool version 2 (RoB 2) was used for bias detection in RCTs (Sterne et al., 2019).

Meta-analysis was performed based on a random-effects model using the restricted maximum likelihood (REML) technique. For continuous variables, data on mean difference (MD) along with its standard deviation (SD) were considered, while for binary variables, the number of events (N) was used. If the outcomes were presented in terms of relative changes (for example, % reduction in UACR), the treatment effect was expressed through the ratio of geometric means (ROM), which was then transformed into natural logarithm [ln(ROM)] to achieve variance stabilization and normality approximation, as advised for ratio-type effect measure in meta-analysis (Friedrich et al., 2012). The standard error (SE) of lnROM was computed based on the 95% confidence intervals (CI) reported in literature. All effect size estimates were combined using an inverse-variance weighting approach and reported along with 95% CI and respective p-values. Heterogeneity was measured by means of the I^2^ statistic (i.e., percentage of variation among study outcomes).

In addition, sensitivity analysis was performed for primary renal outcomes where the pooled estimates showed extreme heterogeneity (>70%) by means of the Baujat plot, influence analysis, and outlier analysis. In particular, the Baujat plot was used to detect the studies that contributed the most to the heterogeneity and overall findings. Influence analysis was conducted via the leave-one-out approach in combination with the standardized residuals. All analyses were carried out in RStudio using the ‘meta’ and ‘metafor’ packages of R statistical software (version 4.5.2) (Posit Team, 2025).

### Certainty of the body of evidence and “summary of findings” tables

The certainty of evidence for each outcome was assessed using the Grading of Recommendations Assessment, Development and Evaluation (GRADE) approach (Meader et al., 2014). The level of evidence was upgraded or downgraded according to five factors: risk of bias, inconsistency, indirectness, imprecision, and publication bias. The risk of bias was evaluated based on the Cochrane RoB 2, while consistency was assessed in terms of heterogeneity (I^2^) and variability in effect estimate. Indirectness assessments measured the appropriateness of the population, interventions, and outcomes, whereas imprecision evaluated confidence intervals and sample size. Publication bias was determined based on a qualitative assessment of the comprehensiveness of literature searching, presence of unpublished or ongoing studies, and risk of selective reporting following the guidelines of GRADE. Finally, the certainty of evidence was graded as high, moderate, low, or very low in structured summary of findings tables.

## Results

### Study selection and characteristics

**FIGURE 1:**
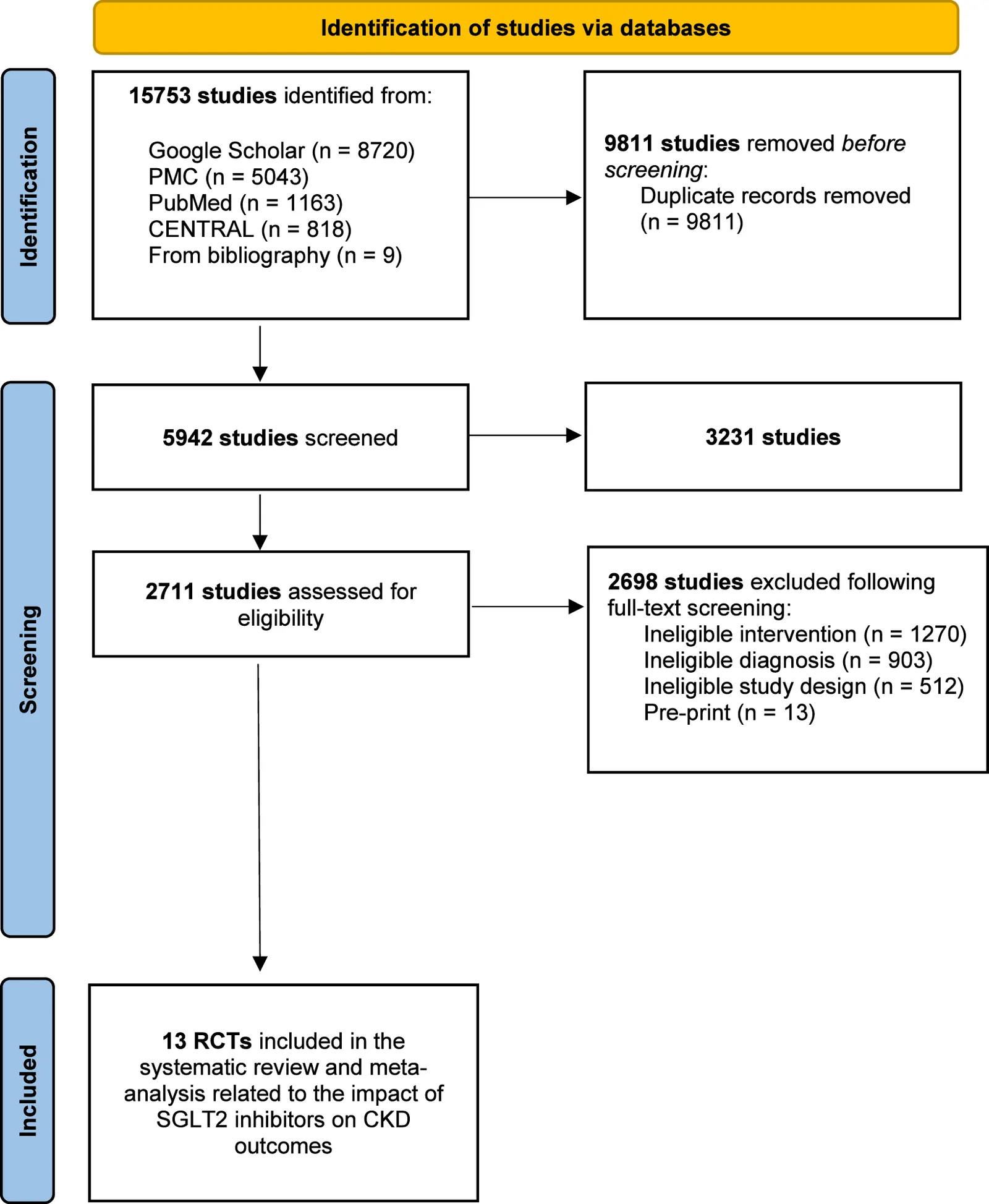
Flow chart showing the methodology of study selection. CENTRAL = Cochrane Central Register of Controlled Trials; CKD = chronic kidney disease; PMC = PubMed Central; SGLT2 = sodium-glucoses co-transporter two.

From the literature search, 15,753 papers were identified. Of these, 5,942 were eligible for title and abstract screening after eliminating duplicate studies (n = 9,811, Figure 1). After excluding 3,231 studies manually, a further analysis of the text was performed for the remaining 2,711 papers. An additional 2,698 studies were excluded due to ineligible interventions (n = 1,270), diagnosis (n = 903), unsuitable study design (n = 512), and preprints (n = 13). Ultimately, a total of 13 RCTs were included in the systematic review and meta-analysis examining the effect of SGLT2 inhibitors on CKD-related parameters.

General features of the 13 RCTs (n = 49,931 subjects), comprising 27,504 subjects under treatment with SGLT2 inhibitors and 22,427 subjects under placebo, are presented in Table 1. Most studies took place across multiple countries, although the study by Bakris et al. (2020) was conducted in the United States, while the study by Wada et al. (2022) took place in Japan. The studies were published between 2013 and 2025.

**TABLE 1:** General characteristics of the included studies and summary of baseline indicators.

Author, primary, year	Study location	Sample size			Female, N (%)	Age, mean ± SD, years	Baseline eGFR, mL/min/1.73 m^2^, Mean ± SD, median (IQR)		Baseline UACR, mg/g, median (IQR)		Follow-up duration, years	Comorbidities	Intervention details		Risk of bias
		Total, N	SGLT2i, N	Placebo, N			SGLT2i	Placebo	SGLT2i	Placebo			SGLT2inhibitor type	Dosage and frequency	
Bakris et al. (2020)	United States	174	84	90	68 (39)	65 ± 10	26 ± 3	27 ± 3	1,056 (459–2,525)	1,153 (483–2,253)	Median = 2.62 (IQR 2.11–3.09)	HTN, CVD, HF, diabetes	Canagliflozin	100 mg once daily	Low
Bhatt et al. (2021)	Multinational (44 countries)	10,584	5,292	5,292	4,754 (44.9)	68.7 ± 8.2	44.4 (37.0–51.3)	44.7 (37.0–51.5)	74 (18–486)	75 (17–477)	Median = 1.33	T2DM	Sotagliflozin	200 mg once daily	Low
Cherney et al. (2021)	Canada, France, Sweden, United Kingdom, United States	8,246	5,499	2,747	2,477 (30.1)	64.4 ± 8.05	76.1 ± 20.9	75.7 ± 20.8	18.0 (6.0–69.0)	19.0 (6.0–66.5)	Median = 3	T2DM, HTN, ASCVD	Ertugliflozin	5 mg or 15 mg once daily	Low
Chertow et al. (2021)	Multinational (21 countries)	624	293	331	209 (33.5)	61.9 ± 12.4	26.8 ± 1.8	26.8 ± 1.8	1,279 (642–2,470)	1,212 (577–2,289)	Median = 2.4	T2DM, CVD, HTN	Dapagliflozin	10 mg once daily	Low
Fioretti et al. (2025)	Multinational (22 countries)	3,033	1,507	1,526	917 (30.2)	65.8 ± 10.5	67.14 ± 21.89	66.83 ± 22.07	-	-	Median = 1.49	HTN	Empagliflozin	10 mg once daily	Low
Heerspink et al. (2020)	Multinational (21 countries)	4,304	2,152	2,152	1,425 (33.1)	61.8 ± 12.1	43.2 ± 12.3	43.0 ± 12.4	965 (472–1903)	934 (482–1868)	Median = 2.4 (IQR 2.0–2.7)	T2DM, CVD, HF, HTN	Dapagliflozin	10 mg once daily	Low
Heerspink et al. (2022)	Brazil, Argentina, India, Canada, Mexico, United Kingdom, United States	1,250	625	625	540 (43.2)	61.1 ± 13.9	84 ± 25	83 ± 25	—	—	30 days	COVID-19, HTN, T2DM, HF, CVD	Dapagliflozin	10 mg once daily	Low
The EMPA-KIDNEY Collaborative Group et al., 2023	Canada, China, Germany, Italy, Japan, Malaysia, United Kingdom, United States	6,609	3,304	3,305	2,193 (33.2)	63.8 ± 13.9	37.4 ± 14.5	37.3 ± 14.4	331 (46–1,061)	327 (54–1,074)	Median = 2	T2DM, CVD, HTN	Empagliflozin	10 mg once daily	Low
Levin et al. (2020)	Multinational (42 countries)	7,020	4,687	2,333	2,036 (29)	67 ± 9	74 ± 20	74 ± 20	43.3 (16.8–94.6)	43.3 (16.8–94.6)	Median = 3.1 (IQR 2.2–3.6)	T2DM, ASCVD, HF	Empagliflozin	10 mg or 25 mg once daily	Low
Perkovic et al. (2019)	Multinational (34 countries)	4,401	2,202	2,199	1,494 (33.9)	63.0 ± 9.2	56.2 ± 18.2	56.0 ± 18.2	927 (463–1833)	931 (463–1833)	Median = 2.62 (IQR 0.02–4.53)	T2DM, HTN, CVD, HF	Canagliflozin	100 mg once daily	Some concerns
Sharma et al. (2023)	Asia, Europe, North America, Latin America	3,198	1,615	1,583	2,674 (44.7)	74.2 ± 8.7	46.3 ± 12.9	46.3 ± 12.9	32 (9–168)	32 (9–168)	Median = 2.18	HF, HTN, diabetes, atrial fibrillation/flutter, obesity	Empagliflozin	10 mg once daily	Low
Wada et al. (2022)	Japan	308	154	154	64 (20.8)	62.5 ± 10.7	56.3 ± 15.5	55.2 ± 13.6	712 (422–1,433)	630 (418–1,239)	2	T2DM, HTN	Canagliflozin	100 mg once daily	Some concerns
Yale et al. (2013)	Multinational (19 countries)	180	90	90	106 (39.4)	68.5 ± 8.3	39.7 ± 6.9	40.1 ± 6.8	Median = 23.7	Median = 31.3	1	T2DM, nephropathy, neuropathy, retinopathy, ASCVD	Canagliflozin	100 mg once daily	Low

Abbreviations: ASCVD, atherosclerotic cardiovascular disease; CVD, cardiovascular disease; HF, heart failure; HTN, hypertension; IQR, interquartile range; MI, myocardial infarction; SD, standard deviation; T2DM, Type 2 Diabetes Mellitus; UACR, Urinary Albumin-to-Creatinine Ratio; USA, united states of america; UK, united kingdom.

In total, the studies included 18,957 females (35.9%). On average, the study population was predominantly older adults with a mean age of 66.3 years (SD = 10.7). The initial kidney functionality was similar between the sample groups: with a mean eGFR of 55.5 mL/min/1.73 m^2^ (SD = 22.7) for SGLT2 inhibitors versus 55.4 mL/min/1.73 m^2^ (SD = 22.5) for placebo. Although kidney filtration rates were comparable, significant differences were observed in UACR, which ranged from a low concentration of protein to severe kidney damage.

In general, most patients had multiple comorbidities, including type 2 diabetes, high blood pressure, and heart failure. Overall, the patients were monitored for approximately 2.4 years, although the duration of the study varied from 1 month to 4 years. The SGLT2 inhibitors included canagliflozin, dapagliflozin, empagliflozin, and sotagliflozin, administered once daily. The data for the effects on the kidneys, heart, and safety of the patients are available in Supplementary Table S1. These outcomes have been pooled for the meta-analysis presented in Table 1.

**FIGURE 2:**
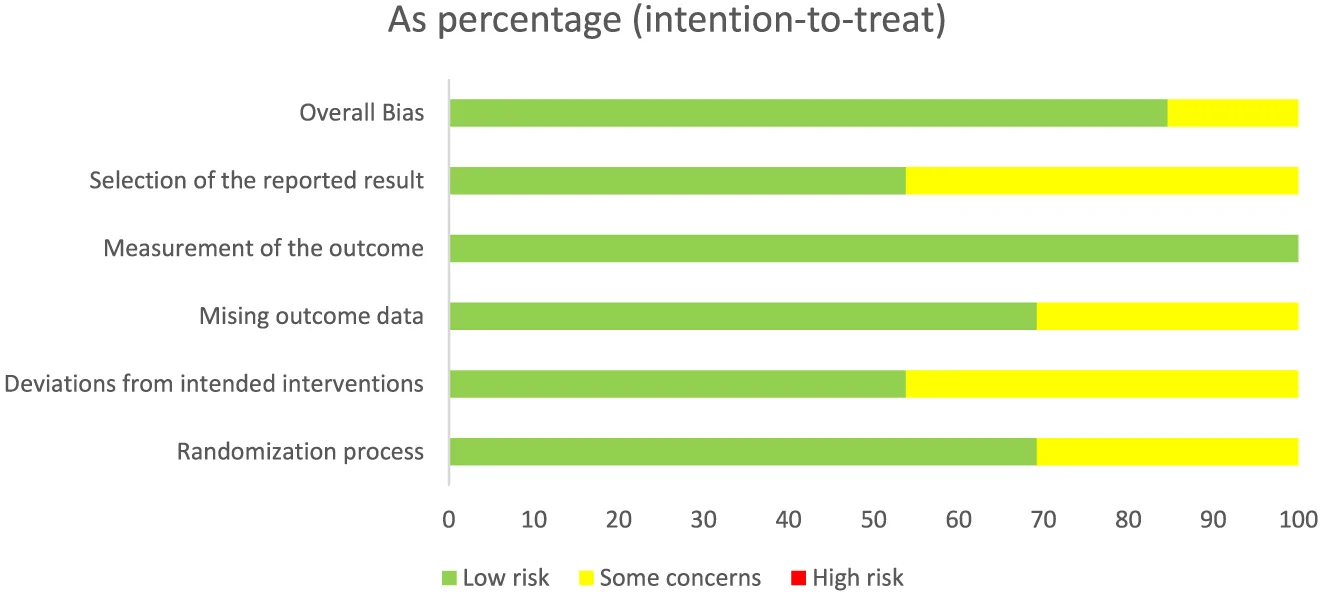
Risk of bias assessment of randomized controlled studies using revised Cochrane Risk-of-Bias tool (RoB 2) (Sterne et al., 2019).

A total of five critical criteria were assessed with RoB 2 for bias evaluation, namely, the randomization process, deviation from intended interventions, missing outcome data, outcome measurement, and outcome reporting, along with overall risk of bias (Figure 2; Supplementary Table S2). The majority of studies had a low overall risk of bias (n = 11), especially with regard to outcomes measurement and reporting (Bakris et al., 2020; Bhatt et al., 2021; Che et al., 2021; Chertow et al., 2021; Fioretti et al., 2025; Heerspink et al., 2020; Heerspink et al., 2022; The EMPA-KIDNEY C ollaborative Group et al., 2023; Levin et al., 2020; Sharma et al., 2023; Yale et al., 2013). However, two of the studies showed potential risk of bias of “some concern,” especially in relation to randomization and the difference between the intended and actual interventions, which may be seen as possible limitations in the methodology used during the research studies (Wada et al., 2022; Perkovic et al., 2019). There were very few occurrences of issues of greater significance, and no studies are deemed to have a high risk of bias. This implies that the quality of evidence is generally good in terms of the domains that were analysed.

### Meta-analysis of the impact of SGLT2 inhibitors on CKD outcomes

**FIGURE 3:**
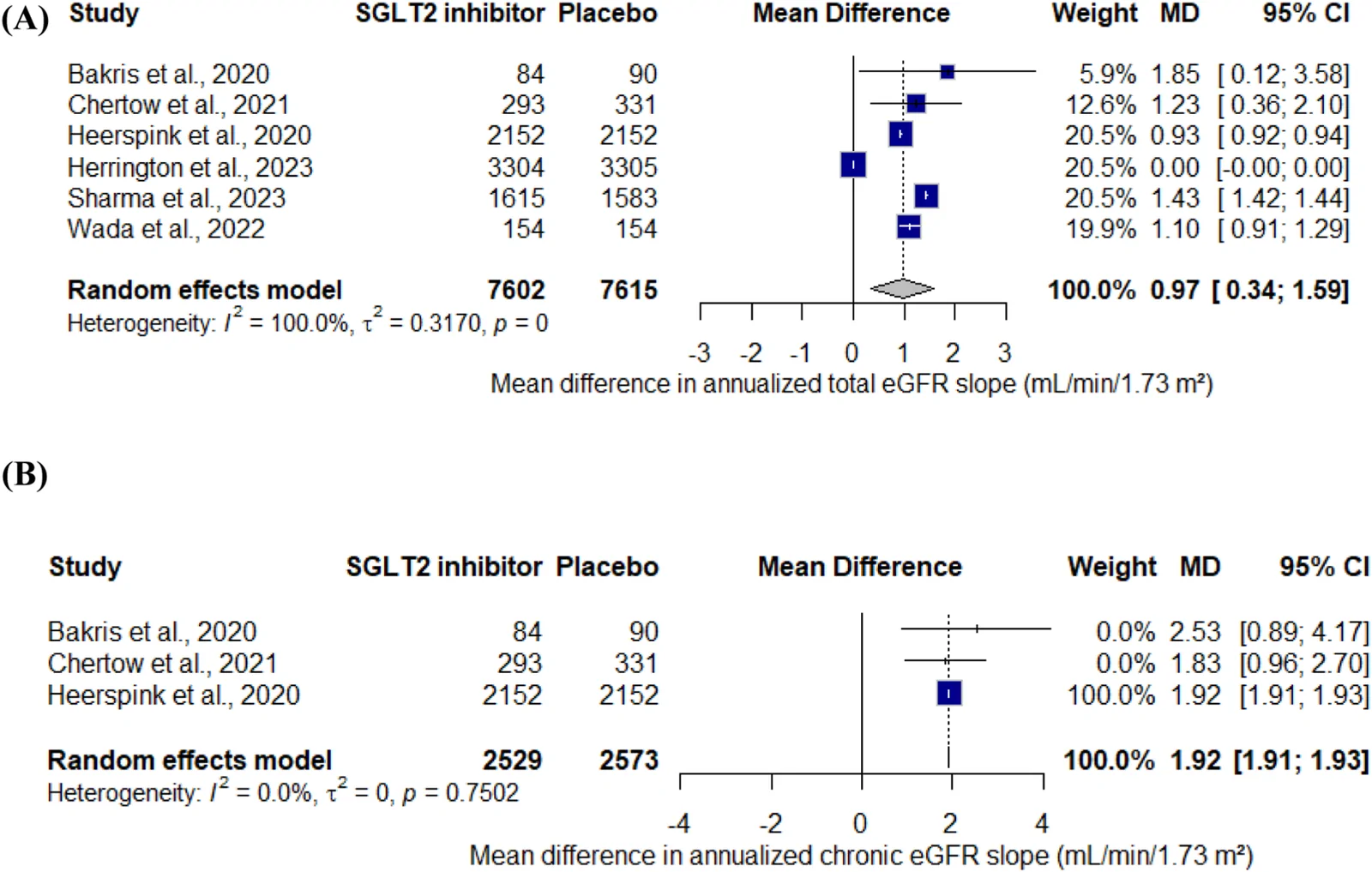
Mean difference in annualized **(A)** total and **(B)** chronic estimated glomerular filtration rate (eGFR, mL/min/1.73 m^2^) among patients treated with an SGLT2 inhibitor vs. placebo.

Meta-analysis using a random effects model was used to assess the MD in annualized eGFR slope. Six RCTs (SGLT2i = 7,602, placebo = 7,615) revealed a large MD in annualized total eGFR slope of 0.97 mL/min/1.73 m^2^ (95% CI: 0.34 to 1.59, I^2^ = 100%, p = < 0.001, Figure 3A). Similarly, three studies (SGLT2i = 2,529, placebo = 2,573) showed statistically significant MD of 1.92 mL/min/1.73 m^2^ (95% CI: 1.91 to 1.93, I^2^ = 0.0%, p = 0.75) pertaining to annualized chronic eGFR slope (Figure 3B).

**FIGURE 4:**
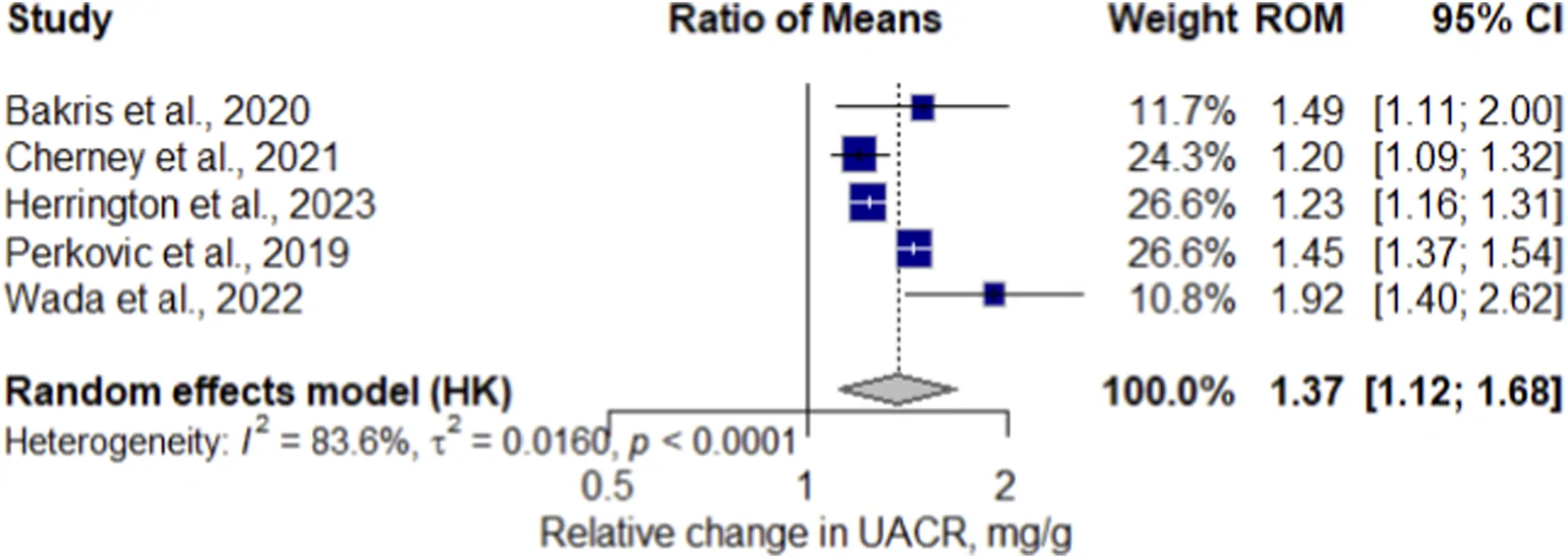
Ratio of means for change in urinary albumin-to-creatinine ratio (UACR, mg/g) with SGLT2 inhibitors vs. placebo.

There were significant reductions in UACR with SGLT2 inhibitors, with a 37% higher relative change in UACR of in the treatment group compared with the control group (95% CI: 1.12 to 1.68, I^2^ = 83.6%, p < 0.0001, Figure 4).

**FIGURE 5:**
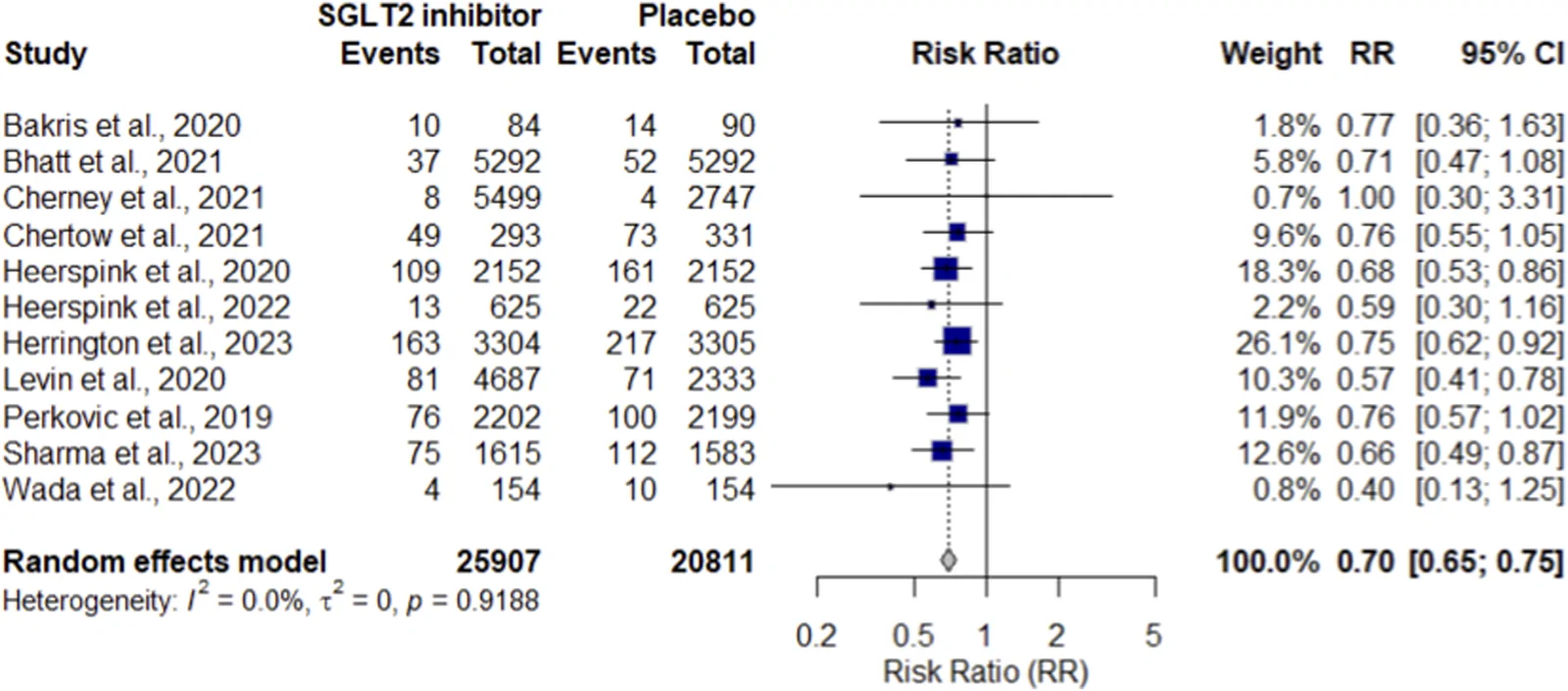
Risk ratios for overall incidence of end-stage kidney disease (ESKD) among patients treated with an SGLT2 inhibitor vs. placebo.

In the pooled analysis using 11 trials (SGLT2i = 25,907, placebo = 20,811), there was a substantial decline in the development of ESKD in patients under SGLT2i treatment relative to the placebo group (RR = 0.70, 95% CI: 0.65 to 0.75, I^2^ = 0.0%, p = 0.92, Figure 5).

**FIGURE 6:**
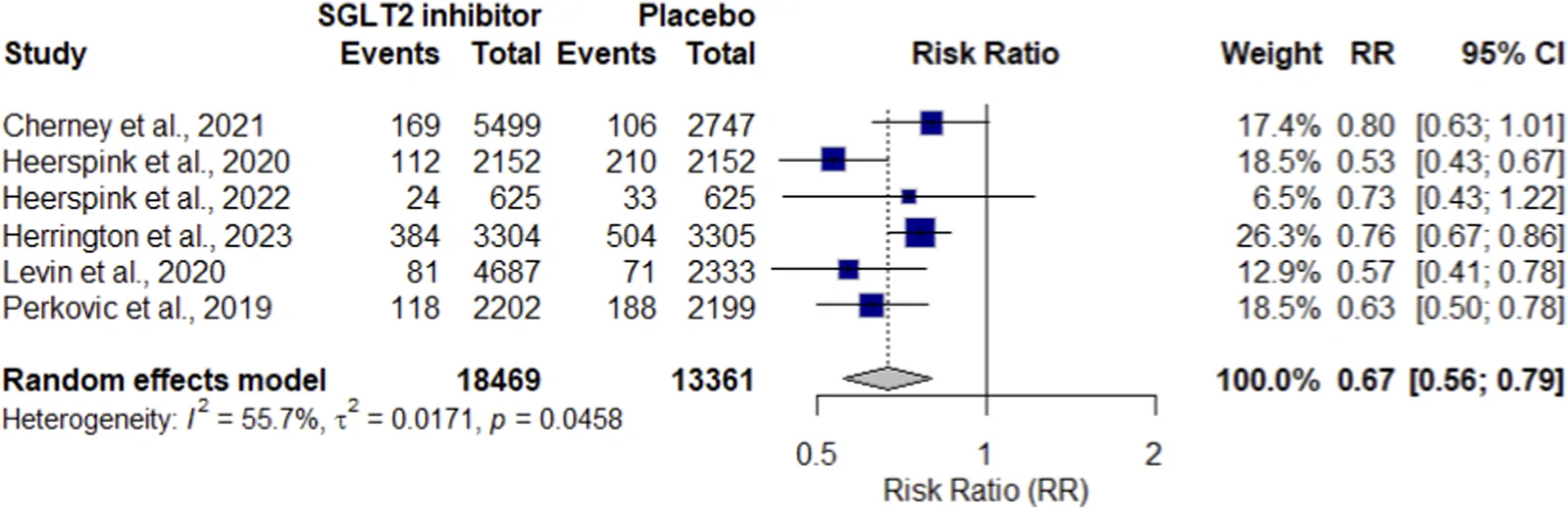
Risk ratios for overall incidence of doubling of serum creatinine among patients on SGLT2 inhibitor vs. placebo.

Likewise, there was a substantial decrease in the rate of serum creatinine doubling with SGLT2 inhibitor therapy versus placebo in six trials with a total of 18,469 patients in the SGLT2 inhibitor cohort and 13,361 patients in the placebo cohort (RR = 0.67, 95% CI: 0.56 to 0.79, I^2^ = 55.7%, p = 0.04, Figure 6).

**FIGURE 7:**
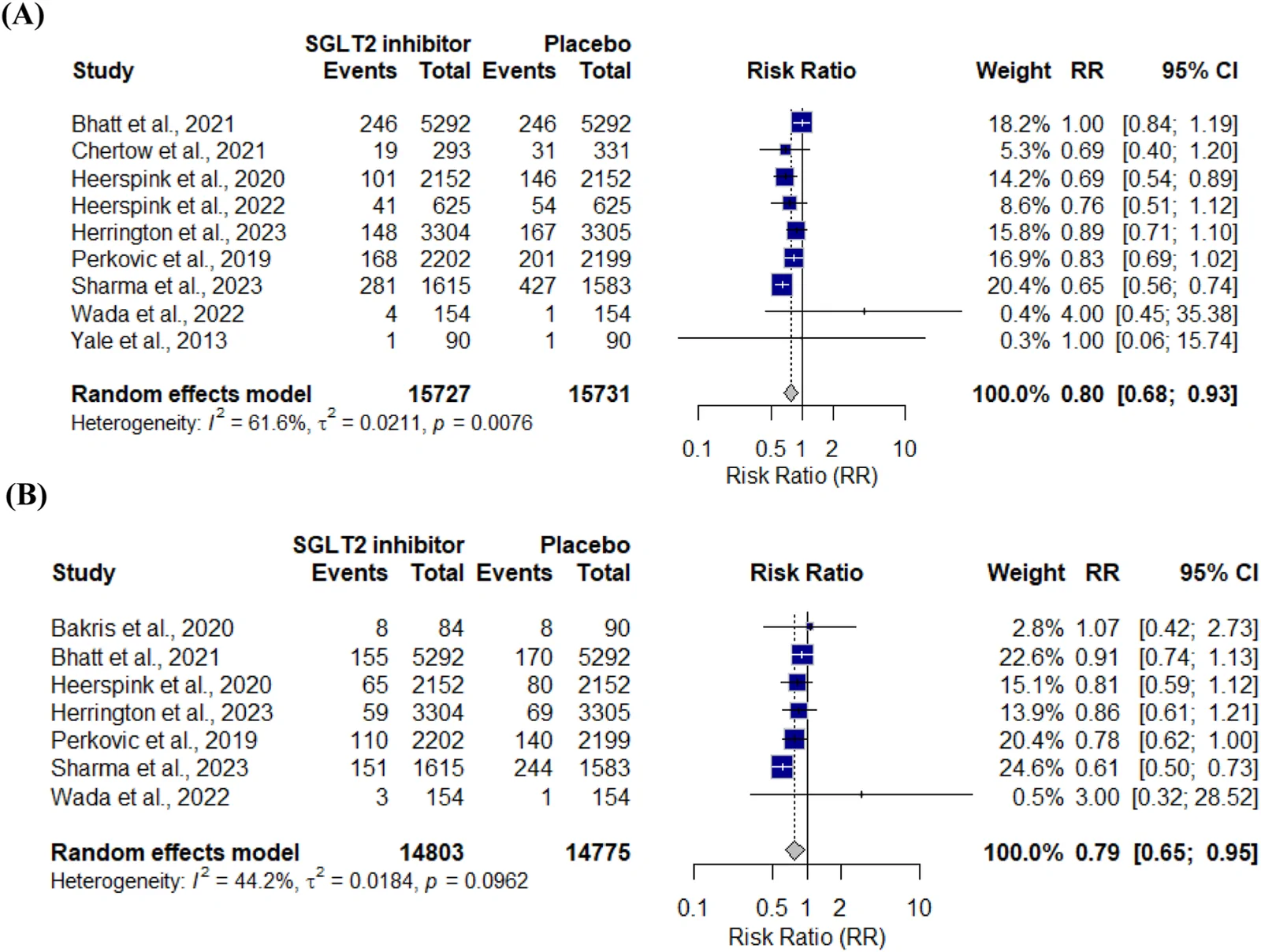
Risk ratios for overall incidence of **(A)** all-cause mortality and **(B)** cardiovascular mortality among patients treated with an SGLT2 inhibitor vs. placebo.

The pooled results obtained from nine studies (SGLT2i = 15,727; placebo = 15,731) and seven studies (SGLT2i = 14,803; placebo = 14,775) indicated that SGLT2 inhibitors were associated with a significant decrease in both all-cause mortality (RR = 0.80, 95% CI: 0.68 to 0.93, I^2^ = 61.6%, p = 0.007) and cardiovascular mortality (RR = 0.79, 95% CI: 0.65 to 0.95, I^2^ = 44.2%, p = 0.09) compared with placebo (Figures 7A,B).

The use of SGLT2 inhibitors was associated with lower odds of hospital admission due to heart failure (RR = 0.74, 95% CI: 0.67 to 0.82, I^2^ = 12.3%, p = 0.33, Supplementary Figure S1) and serious adverse events (RR = 0.92, 95% CI: 0.88 to 0.95, I^2^ = 0.0%, p = 0.54, Supplementary Figure S3), but was not significantly different in relation to overall adverse events (RR = 0.99, 95% CI: 0.96 to 1.02, I^2^ = 21.3%, p = 0.26, Supplementary Figure S2), UTIs (RR = 1.06, 95% CI: 0.99 to 1.13, I^2^ = 0.0%, p = 0.89, Supplementary Figure S5), bone fractures (RR = 1.01, 95% CI: 0.75 to 1.36, I^2^ = 21.0%, p = 0.28, Supplementary Figure S8), kidney-related adverse events (RR = 0.92, 95% CI: 0.69 to 1.22, I^2^ = 23.9%, p = 0.26, Supplementary Figure S9), and amputation (RR = 0.93, 95% CI: 0.57 to 1.52, I^2^ = 0.0%, p = 0.82, Supplementary Figure S10). However, there was a marked increase in occurrence of diabetic ketoacidosis (RR = 2.06, 95% CI: 1.19 to 3.58, I^2^ = 0.0%, p = 0.57, Supplementary Figure S4) and volume depletion (RR = 1.28, 95% CI: 1.13 to 1.45, I^2^ = 0.0%, p = 0.48, Supplementary Figure S6), whereas the number of genital infections was noted to have increased, but not significantly with high heterogeneity (RR = 1.78, 95% CI: 0.78 to 4.09, I^2^ = 75.5%, Supplementary Figure S7).

### Sensitivity analysis

For the pooled analysis of the MD in annualized total eGFR, the Baujat plot identified the study by Herrington et al. as having the greatest influence on the pooled effect size and heterogeneity (Supplementary Figure S11) (The EMPA-KIDNEY C ollaborative Group et al., 2023); however, there were no outliers identified (Supplementary Figure S12), and influence analysis showed that no individual study substantially affected the magnitude or direction of the treatment effect, with statistically significant MDs ranging from 0.85 to 1.18 (Supplementary Figure S13).

Similarly, for the meta-analysis of the ratio of means for change in UACR, the Baujat plot also identified the study by Herrington et al. as having the greatest influence on the pooled effect size and heterogeneity (Supplementary Figure S14) (The EMPA-KIDNEY C ollaborative Group et al., 2023); however, no outliers were found (Supplementary Figure S15), and no individual study was found to substantially influence the magnitude or direction of the treatment effect, with statistically significant RRs ranging from 1.31 to 1.44 (Supplementary Figure S16).

### Certainty of evidence

The certainty of evidence assessed using the GRADE approach was predominantly high to moderate across the evaluated outcomes (Table 2). High-certainty evidence supported the effects on annualized chronic eGFR slope, ESKD, cardiovascular mortality, hospitalization for heart failure, serious adverse events, overall adverse events, diabetic ketoacidosis, urinary tract infections, and volume depletion, reflecting consistent findings, low risk of bias, and precise effect estimates. Moderate-certainty evidence was observed for annualized total eGFR slope, change in UACR, doubling of serum creatinine, all-cause mortality, bone fractures, kidney-related adverse events, and amputation, primarily due to inconsistency or imprecision. Genital infections were supported by low-certainty evidence because of substantial heterogeneity and large confidence intervals crossing the null effect. Overall, the evidence indicates a high level of confidence in most efficacy and safety outcomes, with uncertainty mainly limited to outcomes affected by heterogeneity or imprecise estimates.

**TABLE 2:** Summary of findings using GRADE approach for clinical outcomes (Meader et al., 2014).

Outcome	No. of studies (participants)	Effect estimate (95% CI)	Certainty of evidence (GRADE)	Comments
Annualized total eGFR slope	6 RCTs (15,217 participants)	MD 0.97 (0.34–1.59)	Moderate	Downgraded one level for very serious inconsistency (I^2^ = 100%).
Annualized chronic eGFR slope	3 RCTs (5,102 participants)	MD 1.92 (1.91–1.93)	High	I^2^ = 0% with an extremely precise estimate.
Change in urinary albumin-to-creatinine ratio (UACR)	5 RCTs (19,738 participants)	Ratio of means 1.37 (95% CI 1.12–1.68)	Moderate	Downgraded one level for serious inconsistency (I^2^ = 83.6%).
End-stage kidney disease (ESKD)	11 RCTs (46,718 participants)	RR 0.70 (0.65–0.75)	High	No downgrading. Low risk of bias, consistent findings (I^2^ = 0%), precise estimate.
Doubling of serum creatinine	6 RCTs (31,830 participants)	RR 0.67 (0.56–0.79)	Moderate	Downgraded one level for serious inconsistency (I^2^ = 55.7%).
All-cause mortality	9 RCTs (31,458 participants)	RR 0.80 (0.68–0.93)	Moderate	Downgraded one level for serious inconsistency (I^2^ = 58.3%).
Cardiovascular mortality	7 RCTs (29,578 participants)	RR 0.79 (0.65–0.95)	High	No downgrading. Low heterogeneity (I^2^ = 17.8%) and precise estimate.
Hospitalization for heart failure	10 RCTs (41,481 participants)	RR 0.74 (0.67–0.82)	High	No downgrading. Consistent findings (I^2^ = 12.3%).
Serious adverse events	10 RCTs (28,056 participants)	RR 0.92 (0.88–0.95)	High	No downgrading. No heterogeneity (I^2^ = 0%).
Overall adverse events	7 RCTs (15,598 participants)	RR 0.99 (0.96–1.02)	High	No downgrading. Low heterogeneity (I^2^ = 21.3%).
Diabetic ketoacidosis	8 RCTs (31,113 participants)	RR 2.06 (1.19–3.58)	High	No downgrading. Consistent findings (I^2^ = 0%).
Urinary tract infections	5 RCTs (14,894 participants)	RR 1.06 (0.99–1.13)	High	No downgrading. No heterogeneity (I^2^ = 0%); confidence interval narrowly includes no effect.
Volume depletion	6 RCTs (28,036 participants)	RR 1.28 (1.13–1.45)	High	No downgrading. No heterogeneity (I^2^ = 0%).
Genital infections	6 RCTs (23,912 participants)	RR 1.78 (0.78–4.09)	Low	Downgraded one level for serious inconsistency (I^2^ = 75.5%) and one level for serious imprecision (wide CI crossing no effect).
Bone fractures	4 RCTs (18,394 participants)	RR 1.01 (0.75–1.36)	Moderate	Downgraded one level for imprecision (wide CI crossing no effect).
Kidney-related adverse events	4 RCTs (18,545 participants)	RR 0.92 (0.69–1.22)	Moderate	Downgraded one level for imprecision (wide CI crossing no effect).
Amputation	2 RCTs (14,888 participants)	RR 0.93 (0.57–1.52)	Moderate	Downgraded one level for serious imprecision (wide CI crossing important benefit and harm).

## Discussion

The current systematic review and meta-analysis included data from 13 RCTs comprising 49,931 patients with CKD to assess the effectiveness and safety profile of SGLT2 inhibitors about a broad range of kidney and cardiac endpoints. The combined results show that SGLT2 inhibitors have a beneficial effect on the course of CKD, demonstrated by statistically significant positive changes in the total and chronic eGFR slopes, a dramatic reduction in the UACR, as well as considerable decreases in the rate of ESKD and serum creatinine doubling. Besides renal benefits, the use of SGLT2 inhibitors resulted in a significant decline in the risk of all-cause mortality, cardiovascular mortality, and hospitalization for heart failure, highlighting the importance of using SGLT2 inhibitors as the backbone of cardiorenal prevention strategies. Thus, our study expands the potential therapeutic profile of SGLT2 inhibitors beyond the findings from landmark RCTs such as Dapagliflozin and Prevention of Adverse outcomes in chronic kidney disease (DAPA-CKD), Canagliflozin and Renal Events in Diabetes with Established Nephropathy Clinical Evaluation (CREDENCE), and EMPA-KIDNEY studies.

The increase in the total eGFR slope by SGLT2 is significant because every point saved in the eGFR slope per year corresponds to a lowered progression towards ESKD and avoiding the need for renal replacement therapy (Heerspink and Cherney, 2021). The broad confidence interval and high heterogeneity (I^2^ = 100%) observed in our analysis probably arise from different follow-up times, initial eGFR levels, and albuminuria levels among trials rather than a lack of effect. The evidence is reinforced by the substantial and consistent decline in UACR levels, with SGLT2 inhibitors exhibiting a greater relative decrease in UACR levels compared to placebo (Fengjun et al., 2025). It has been hypothesized that antiproteinuria observed with SGLT2 inhibitors stems from their capacity to decrease intraglomerular hypertension via restoring tubuloglomerular feedback, resulting in glomerular hypofiltration and sparing of podocytes (Vargas-Brochero et al., 2026).

A 30% decrease in the relative risk of ESKD is certainly one of the strongest results from this meta-analysis, considering the total lack of heterogeneity in statistics (I^2^ = 0.0%). This is quite an important aspect since the prevention of ESKD holds significant importance in terms of patient quality of life, the use of medical resources, and morbidity/mortality related to dialysis therapy (Doan et al., 2024). Clinical trials such as DAPA-CKD and CREDENCE have also confirmed that SGLT2 inhibition prevents the progression of CKD and lowers the risk of developing ESKD regardless of the presence of type 2 diabetes (Zhang and Deng, 2025; Mende, 2022). There was a substantial decrease of 33% in the risk of doubling serum creatinine levels by SGLT2 inhibition treatment, commonly used marker of irreversible loss of nephrons (Takeuchi et al., 2011). This is comparable to the reports from previous meta-analysis studies which showed that SGLT2 inhibition decreases the risk of kidney disease progression, including the doubling of serum creatinine levels, by 33%–37% (Baigent et al., 2022).

The results of the meta-analysis regarding mortality are especially interesting. SGLT2 inhibitors were associated with lower all-cause mortality and lower cardiovascular mortality. These are the results that are most clinically significant, given that cardiovascular diseases are the major cause of mortality in the target population. The low heterogeneity associated with the reduction in cardiovascular mortality (I^2^ = 44.2%) in contrast to the higher heterogeneity associated with all-cause mortality (I^2^ = 61.6%) indicates that the cardiovascular mortality benefit is relatively homogeneous across studies, whereas the heterogeneity associated with all-cause mortality may be attributed to variations in baseline morbidity, other causes of mortality besides cardiovascular events, and the duration of follow-up. The decline in heart failure hospitalizations by 27% is consistent with the well-understood mechanisms of action, including the mediation of diuresis by SGLT2 inhibitors, preload and afterload lowering, and change in metabolism favoring usage of ketones as fuel for the myocardium, improving the energy yield (Palmiero et al., 2021). Therefore, the dual cardiorenal protection demonstrated by these medications reflects their pleiotropic effects on the body.

In terms of safety, the general adverse events profile for SGLT2 inhibitors was mostly positive. There were no major differences in overall adverse events, UTIs, bone fractures, renal adverse events, or amputations. Yet, there are two safety alerts that require consideration. First, there was a two-fold higher risk of developing DKA. This is aligned with the drug’s pharmacology, since SGLT2 inhibitors induce ketone body production regardless of glucose levels in the blood, potentially made worse by lower insulin levels or dietary restriction (Blau et al., 2017). Despite the overall low risk of DKA in the trials considered herein, clinicians must be more cautious in patients with inadequate insulin levels, fasting, or undergoing surgery. Likewise, an increase of 30% in the number of cases of volume depletion is due to the diuretic and natriuretic effects of this medication class, which, although helpful in conditions with fluid excess like heart failure, can lead to hypotension in patients with severe CKD, especially when combined with antihypertensive drugs (Novak and Ellison, 2022). Genital mycotic infections had a higher risk without being statistically significant.

Several caveats regarding this meta-analysis should be noted. Firstly, although the analysis incorporated patients with and without diabetes, most of the participants in the included trials had type 2 diabetes, which may constrain the extrapolation of the findings to non-diabetic CKD populations. Secondly, considerable heterogeneity was observed for total eGFR slope and UACR, which may be attributable to clinical differences across studies, including variation in CKD stage, baseline kidney function, baseline albuminuria, duration of follow-up, and participant characteristics, rather than methodological shortcomings alone. In addition, the included trials evaluated different SGLT2 inhibitors (canagliflozin, dapagliflozin, empagliflozin, ertugliflozin, and sotagliflozin), and although these agents are mechanistically similar, potential differences in their pharmacologic profiles and trial populations may have contributed to between-study variability. Finally, the average follow-up duration of approximately 2.4 years may be inadequate to fully evaluate the long-term durability of renal protection and structural kidney outcomes. Therefore, the pooled estimates, particularly for eGFR slope and albuminuria, should be interpreted with appropriate caution.

Clinically and as part of health policy, the results obtained through this meta-analysis support the use of SGLT2 inhibitors as an appropriate choice for first-line management of CKD patients, regardless of whether they are diabetic, consistent with nephrology and cardiology clinical guidelines. The observed renal and cardiac benefits with all tested SGLT2 inhibitors (canagliflozin, dapagliflozin, empagliflozin, and sotagliflozin), supports a potential class effect, with flexibility in choice of intervention dictated by patient specifics and costs. Further scientific investigations are needed to address the efficacy of individual agents compared to each other, SGLT2 inhibitor combinations with other nephro-protectants like finerenone, and efficacy in various sub-populations like non-diabetic CKD, and patients representing geographic/ethnic minorities currently under-represented in trials.

## Conclusion

This meta-analysis highlights the positive effects of SGLT2 inhibitors on CKD patients in terms of renal and cardiovascular outcomes. The drugs effectively delay the progression of CKD, demonstrated by the slowed rates of eGFR decrease, reduced levels of albuminuria, and lower chances of developing end-stage renal disease and serum creatinine doubling. Moreover, they offer significant survival advantages, such as lower mortality due to all causes and cardiovascular diseases, as well as reduced hospitalization due to heart failure. While SGLT2 inhibitors have an overall favorable safety profile, specific adverse events require appropriate clinical monitoring and thus healthcare professionals need to be careful about the increased likelihood of DKA and volume depletion.

## Data Availability

The original contributions presented in the study are included in the article/Supplementary Material, further inquiries can be directed to the corresponding authors.

## References

[B1] Baigent C. Emberson J. R. Haynes R. Herrington W. G. Judge P. Landray M. J. (2022). Impact of diabetes on the effects of sodium glucose co-transporter-2 inhibitors on kidney outcomes: collaborative meta-analysis of large placebo-controlled trials. Lancet 400, 1788–1801. 10.1016/S0140-6736(22)02074-8 36351458 PMC7613836

[B2] Bakris G. Oshima M. Mahaffey K. W. Agarwal R. Cannon C. P. Capuano G. (2020). Effects of Canagliflozin in patients with baseline eGFR <30 ml/min per 1.73 m^2^: subgroup analysis of the randomized CREDENCE trial. Clin. J. Am. Soc. Nephrol. 15, 1705–1714. 10.2215/CJN.10140620 33214158 PMC7769025

[B3] Bhatt D. L. Szarek M. Pitt B. Cannon C. P. Leiter L. A. McGuire D. K. (2021). Sotagliflozin in patients with diabetes and chronic kidney disease. N. Engl. J. Med. 384, 129–139. 10.1056/NEJMoa2030186 33200891

[B4] Blau J. E. Tella S. H. Taylor S. I. Rother K. I. (2017). Ketoacidosis associated with SGLT2 inhibitor treatment: analysis of FAERS data. Diabetes Metab. Res. 33, e2924. 10.1002/dmrr.2924 28736981 PMC5950709

[B5] Caruso I. Giorgino F. (2022). SGLT-2 inhibitors as cardio-renal protective agents. Metabolism 127, 154937. 10.1016/j.metabol.2021.154937 34808144

[B6] Cherney D. Z. I. Charbonnel B. Cosentino F. Dagogo-Jack S. McGuire D. K. Pratley R. (2021). Effects of ertugliflozin on kidney composite outcomes, renal function and albuminuria in patients with type 2 diabetes mellitus: an analysis from the randomised VERTIS CV trial. Diabetologia 64, 1256–1267. 10.1007/s00125-021-05407-5 33665685 PMC8099851

[B7] Chen T. K. Knicely D. H. Grams M. E. (2019). Chronic kidney disease diagnosis and management: a review. JAMA 322, 1294–1304. 10.1001/jama.2019.14745 31573641 PMC7015670

[B8] Chertow G. M. Vart P. Jongs N. Toto R. D. Gorriz J. L. Hou F. F. (2021). Effects of dapagliflozin in stage 4 chronic kidney disease. J. Am. Soc. Nephrol. 32, 2352–2361. 10.1681/ASN.2021020167 34272327 PMC8729835

[B9] Doan V. Shoker A. Abdelrasoul A. (2024). Quality of life of dialysis patients: exploring the influence of membrane hemocompatibility and dialysis practices on psychosocial and physical symptoms. J. Compos Sci. 8, 172. 10.3390/jcs8050172

[B10] Fengjun D. Junli T. Zilong L. Xiaorong C. Jiyu Z. Jianwei X. (2025). Joint association of urinary albumin-to-creatinine ratio within normal range and triglyceride-glucose index with incident cardiovascular disease: a prospective cohort study. Cardiovasc Diabetol. 24, 451. 10.1186/s12933-025-03009-8 41316179 PMC12664133

[B11] Fioretti F. Butler J. Udell J. A. Schuyler Jones W. Petrie M. C. Harrington J. (2025). Empagliflozin after myocardial infarction with or without diabetes and chronic kidney disease: insights from EMPACT-MI. ESC Heart Fail. 12, 3940–3952. 10.1002/ehf2.15393 40947857 PMC12719805

[B12] Friedrich J. O. Adhikari N. K. J. Beyene J. (2012). Ratio of geometric means to analyze continuous outcomes in meta-analysis: comparison to mean differences and ratio of arithmetic means using empiric data and simulation. Statistics Med. 31, 1857–1886. 10.1002/sim.4501 22438170

[B13] Gong J. Vaduganathan M. Wadhera R. K. (2026). Chronic kidney disease prevalence and awareness among US adults. JAMA Cardiol. 11, 77–81. 10.1001/jamacardio.2025.4581 41205219 PMC12596746

[B14] Heerspink H. J. L. Cherney D. Z. I. (2021). Clinical implications of an acute dip in eGFR after SGLT2 inhibitor initiation. Clin. J. Am. Soc. Nephrol. 16, 1278–1280. 10.2215/CJN.02480221 33879500 PMC8455037

[B15] Heerspink H. J. L. Stefánsson B. V. Correa-Rotter R. Chertow G. M. Greene T. Hou F.-F. (2020). Dapagliflozin in patients with chronic kidney disease. N. Engl. J. Med. 383, 1436–1446. 10.1056/NEJMoa2024816 32970396

[B16] Heerspink H. J. L. Furtado R. H. M. Berwanger O. Koch G. G. Martinez F. Mukhtar O. (2022). Dapagliflozin and kidney outcomes in hospitalized patients with COVID-19 infection: an analysis of the DARE-19 randomized controlled trial. Clin. J. Am. Soc. Nephrol. 17, 643–654. 10.2215/CJN.14231021 35483733 PMC9269587

[B17] Kaze A. D. Zhuo M. Kim S. C. Patorno E. Paik J. M. (2022). Association of SGLT2 inhibitors with cardiovascular, kidney, and safety outcomes among patients with diabetic kidney disease: a meta-analysis. Cardiovasc Diabetol. 21, 47. 10.1186/s12933-022-01476-x 35321742 PMC9491404

[B18] Leoncini G. Viazzi F. De Cosmo S. Russo G. Fioretto P. Pontremoli R. (2020). Blood pressure reduction and RAAS inhibition in diabetic kidney disease: therapeutic potentials and limitations. J. Nephrol. 33, 949–963. 10.1007/s40620-020-00803-3 32681470 PMC7557495

[B19] Levin A. Perkovic V. Wheeler D. C. Hantel S. George J. T. Von Eynatten M. (2020). Empagliflozin and cardiovascular and kidney outcomes across KDIGO risk categories: post hoc analysis of a randomized, double-blind, placebo-controlled, multinational trial. Clin. J. Am. Soc. Nephrol. 15, 1433–1444. 10.2215/CJN.14901219 32994159 PMC7536760

[B20] Mark P. B. Stafford L. K. Grams M. E. Aalruz H. Abd ElHafeez S. Abdelgalil A. A. (2025). Global, regional, and national burden of chronic kidney disease in adults, 1990–2023, and its attributable risk factors: a systematic analysis for the global burden of disease study 2023. Lancet 406, 2461–2482. 10.1016/S0140-6736(25)01853-7 41213283

[B21] Meader N. King K. Llewellyn A. Norman G. Brown J. Rodgers M. (2014). A checklist designed to aid consistency and reproducibility of GRADE assessments: development and pilot validation. Syst. Rev. 3, 82. 10.1186/2046-4053-3-82 25056145 PMC4124503

[B22] Mende C. W. (2022). Chronic kidney disease and SGLT2 inhibitors: a review of the evolving treatment landscape. Adv. Ther. 39, 148–164. 10.1007/s12325-021-01994-2 34846711 PMC8799531

[B23] Menne J. Dumann E. Haller H. Schmidt B. M. W. (2019). Acute kidney injury and adverse renal events in patients receiving SGLT2-inhibitors: a systematic review and meta-analysis. PLoS Med. 16, e1002983. 10.1371/journal.pmed.1002983 31815931 PMC6901179

[B24] Moher D. Liberati A. Tetzlaff J. Altman D. G., and PRISMA Group (2010). Preferred reporting items for systematic reviews and meta-analyses: the PRISMA statement. Int. J. Surg. 8, 336–341. 10.1016/j.ijsu.2010.02.007 20171303

[B25] Novak J. E. Ellison D. H. (2022). Diuretics in states of volume overload: Core curriculum 2022. Am. J. Kidney Dis. 80, 264–276. 10.1053/j.ajkd.2021.09.029 35190215

[B26] Palmiero G. Cesaro A. Vetrano E. Pafundi P. C. Galiero R. Caturano A. (2021). Impact of SGLT2 inhibitors on heart failure: from pathophysiology to clinical effects. Int. J. Mol. Sci. 22, 5863. 10.3390/ijms22115863 34070765 PMC8199383

[B27] Perkovic V. Jardine M. J. Neal B. Bompoint S. Heerspink H. J. L. Charytan D. M. (2019). Canagliflozin and renal outcomes in type 2 diabetes and nephropathy. N. Engl. J. Med. 380, 2295–2306. 10.1056/NEJMoa1811744 30990260

[B28] Posit team (2025). RStudio: integrated development environment for R. Available online at: http://www.posit.co/. (Accessed June 25, 2026)

[B29] Salvatore T. Galiero R. Caturano A. Rinaldi L. Di Martino A. Albanese G. (2022). An overview of the cardiorenal protective mechanisms of SGLT2 inhibitors. IJMS 23, 3651. 10.3390/ijms23073651 35409011 PMC8998569

[B30] Sharma A. Ferreira J. P. Zannad F. Pocock S. J. Filippatos G. Pfarr E. (2023). Cardiac and kidney benefits of empagliflozin in heart failure across the spectrum of kidney function: insights from the EMPEROR-preserved trial. Eur. J. Heart Fail. 25, 1337–1348. 10.1002/ejhf.2857 37062851

[B31] Sterne J. A. C. Savović J. Page M. J. Elbers R. G. Blencowe N. S. Boutron I. (2019). RoB 2: a revised tool for assessing risk of bias in randomised trials. BMJ 366, l4898. 10.1136/bmj.l4898 31462531

[B32] Takeuchi N. Takenoshita E. Kato F. Terajima T. Ogawa M. Suzuki S. (2011). Doubling of serum creatinine: is it appropriate as the endpoint for CKD? Proposal of a new surrogate endpoint based on the reciprocal of serum creatinine. Clin. Exp. Nephrol. 15, 100–107. 10.1007/s10157-010-0365-1 21058043

[B33] The EMPA-KIDNEY Collaborative Group., Herrington W. G. Staplin N. Wanner C. Green J. B. Hauske S. J. (2023). Empagliflozin in patients with chronic kidney disease. N. Engl. J. Med. 388, 117–127. 10.1056/NEJMoa2204233 36331190 PMC7614055

[B34] Vallon V. Verma S. (2021). Effects of SGLT2 inhibitors on kidney and cardiovascular function. Annu. Rev. Physiol. 83, 503–528. 10.1146/annurev-physiol-031620-095920 33197224 PMC8017904

[B35] Vargas-Brochero M. J. Russo I. Mazzierli T. Cara A. Berti G. M. Milheiro J. (2026). Antiproteinuric effect of SGLT2 inhibitors in non-diabetic glomerulopathies is dependent on body mass index. Nephrol. Dial. Transplant. 41, 703–714. 10.1093/ndt/gfaf197 40996459 PMC13037471

[B36] Wada T. Mori‐Anai K. Takahashi A. Matsui T. Inagaki M. Iida M. (2022). Effect of canagliflozin on the decline of estimated glomerular filtration rate in chronic kidney disease patients with type 2 diabetes mellitus: a multicenter, randomized, double-blind, placebo-controlled, parallel-group, phase III study in Japan. J. Diabetes Invest. 13, 1981–1989. 10.1111/jdi.13888 35861630 PMC9720226

[B37] Yale J.-F. Bakris G. Cariou B. Yue D. David-Neto E. Xi L. (2013). Efficacy and safety of canagliflozin in subjects with type 2 diabetes and chronic kidney disease. Diabetes Obes. Metab. 15, 463–473. 10.1111/dom.12090 23464594 PMC3654568

[B38] Zelniker T. A. Wiviott S. D. Raz I. Im K. Goodrich E. L. Bonaca M. P. (2019). SGLT2 inhibitors for primary and secondary prevention of cardiovascular and renal outcomes in type 2 diabetes: a systematic review and meta-analysis of cardiovascular outcome trials. Lancet 393, 31–39. 10.1016/S0140-6736(18)32590-X 30424892

[B39] Zhang B. Deng L. (2025). Application of SGLT-2 inhibitors in non-diabetic CKD: mechanisms, efficacy, and safety. Front. Med. 12, 1574693. 10.3389/fmed.2025.1574693 40665981 PMC12259609

[B40] Zhang F. Liu H. Liu D. Liu Y. Li H. Tan X. (2017). Effects of RAAS inhibitors in patients with kidney disease. Curr. Hypertens. Rep. 19, 72. 10.1007/s11906-017-0771-9 28791529

[B41] Zoccali C. Mallamaci F. Adamczak M. De Oliveira R. B. Massy Z. A. Sarafidis P. (2023). Cardiovascular complications in chronic kidney disease: a review from the European Renal and Cardiovascular Medicine Working Group of the European Renal Association. Cardiovasc. Res. 119, 2017–2032. 10.1093/cvr/cvad083 37249051 PMC10478756

