# Supplementary material for: Effectiveness of SGLT2 inhibitors in slowing chronic kidney disease progression and reducing mortality: a meta-analysis
Source: Front Pharmacol. 2026 Aug 31;17:1923548. doi: 10.3389/fphar.2026.1923548 (PMC13572723; doi:10.3389/fphar.2026.1923548)
Supplement: Supplementary file 1 [file DataSheet1.pdf]

Appendix A

Search Strings and MeSH Terms

Database	Search Strategy
**CENTRAL**	(CKD OR "chronic kidney disease" OR "chronic renal disease" OR "chronic kidney failure" OR "renal insufficiency" OR "kidney dysfunction") AND ("SGLT2 inhibitor*" OR "sodium-glucose cotransporter 2 inhibitor*" OR empagliflozin OR dapagliflozin OR canagliflozin OR ertugliflozin) AND (progression OR "disease progression" OR decline OR "kidney function" OR eGFR OR "end-stage kidney disease" OR ESKD OR mortality OR death)
**Google Scholar**	"SGLT2 inhibitors" AND "chronic kidney disease" AND ("eGFR decline" OR "end-stage kidney disease" OR mortality) AND ("randomized controlled trial" OR RCT)
**PMC**	("Chronic Kidney Disease" OR CKD) AND ("SGLT2 Inhibitors" OR empagliflozin OR dapagliflozin) AND ("eGFR decline" OR "End-Stage Kidney Disease" OR mortality) AND ("Randomized Controlled Trial" OR RCT)
**PubMed**	("Chronic Kidney Disease"[MeSH] OR "Renal Insufficiency, Chronic"[MeSH] OR CKD OR "chronic kidney disease") AND ("Sodium-Glucose Transporter 2 Inhibitors"[MeSH] OR "SGLT2 inhibitors" OR empagliflozin OR dapagliflozin OR canagliflozin OR ertugliflozin) AND ("Disease Progression"[MeSH] OR "Kidney Failure, Chronic"[MeSH] OR "Glomerular Filtration Rate"[MeSH] OR mortality OR death)

Appendix B

PICOS Criteria Used for Study Selection

**Population**	adult patients (≥18 years) diagnosed with chronic kidney disease (CKD), including both diabetic and non-diabetic CKD populations, across all stages
**Intervention**	administration of sodium-glucose cotransporter 2 (SGLT2) inhibitors, including but not limited to empagliflozin, dapagliflozin, canagliflozin, and ertugliflozin, at any approved dose and duration
**Comparison**	placebo
**Outcomes**	total and chronic estimated glomerular filtration rate (eGFR) slope, relative change in urine albumin-to-creatinine ratio (UACR), incidence of end-stage kidney disease (ESKD) (progression to dialysis or transplant), doubling of serum creatinine, heart failure admission, all-cause death, cardiovascular death, adverse events, serious adverse events, specific side-effects [diabetic ketoacidosis (DKA), urinary tract infection (UTI), volume depletion, genital infection, bone fracture, amputation, and renal side effects]
**Study Designs**	randomized controlled trials (RCTs)
**Timing**	short-term (< 12 weeks) or long-term (≥ 12 weeks) studies
**Setting**	hospital-based, outpatient, and community settings across all geographic regions

Appendix C

Eligibility Criteria

Inclusion Criteria	Exclusion Criteria
• adult patients (≥18 years) diagnosed with chronic kidney disease (CKD)<br>• studies evaluating SGLT2 inhibitors as a primary intervention, and reporting CKD progression, renal outcomes, or mortality<br>• peer-reviewed articles available in English language	• pediatric populations; patients without CKD, or studies focusing solely on acute kidney injury (AKI)<br>• studies not involving SGLT2 inhibitors or combining interventions where SGLT2-specific effects cannot be isolated<br>• animal/in vitro studies<br>• duplicate publications, editorials, reviews, or opinion pieces without complete data

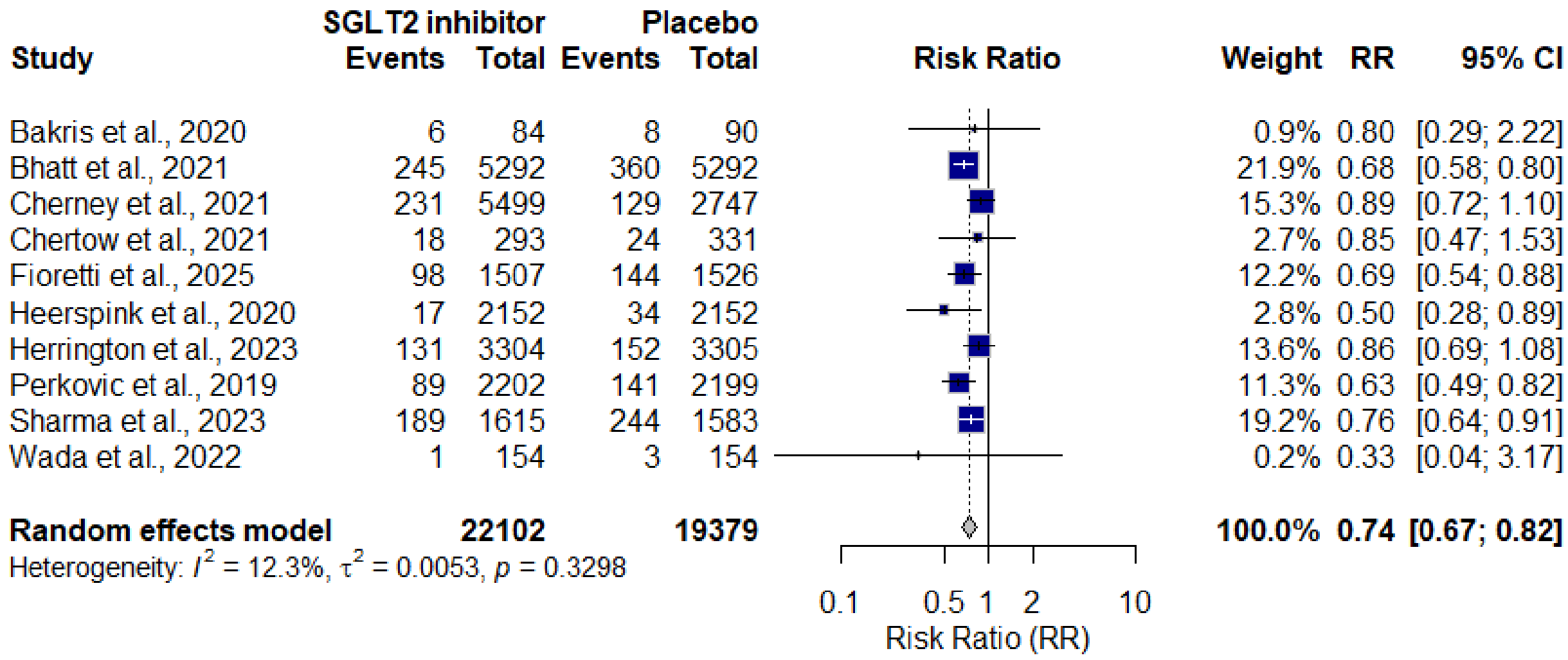

Supplementary Figure S1. Risk ratios for overall incidence of hospitalization for heart failure among patients treated with an SGLT2 inhibitor vs. placebo

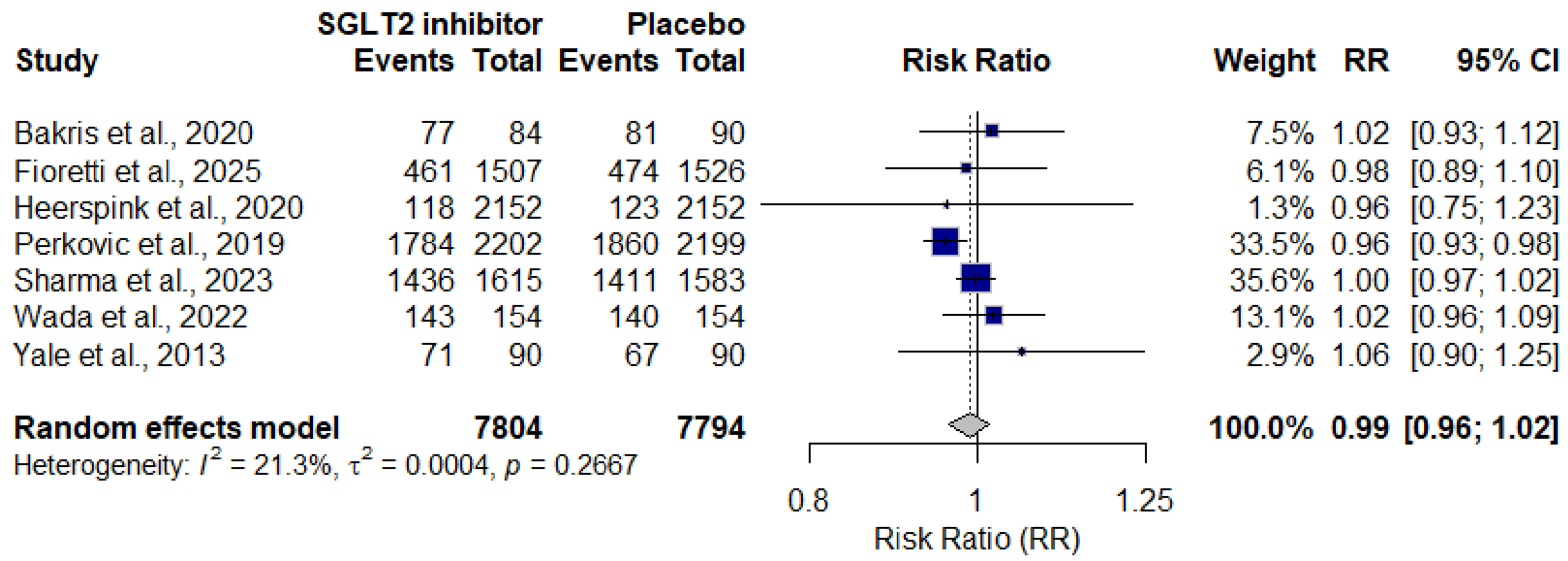

Supplementary Figure S2. Risk ratios for overall incidence of adverse events among patients treated with an SGLT2 inhibitor vs. placebo

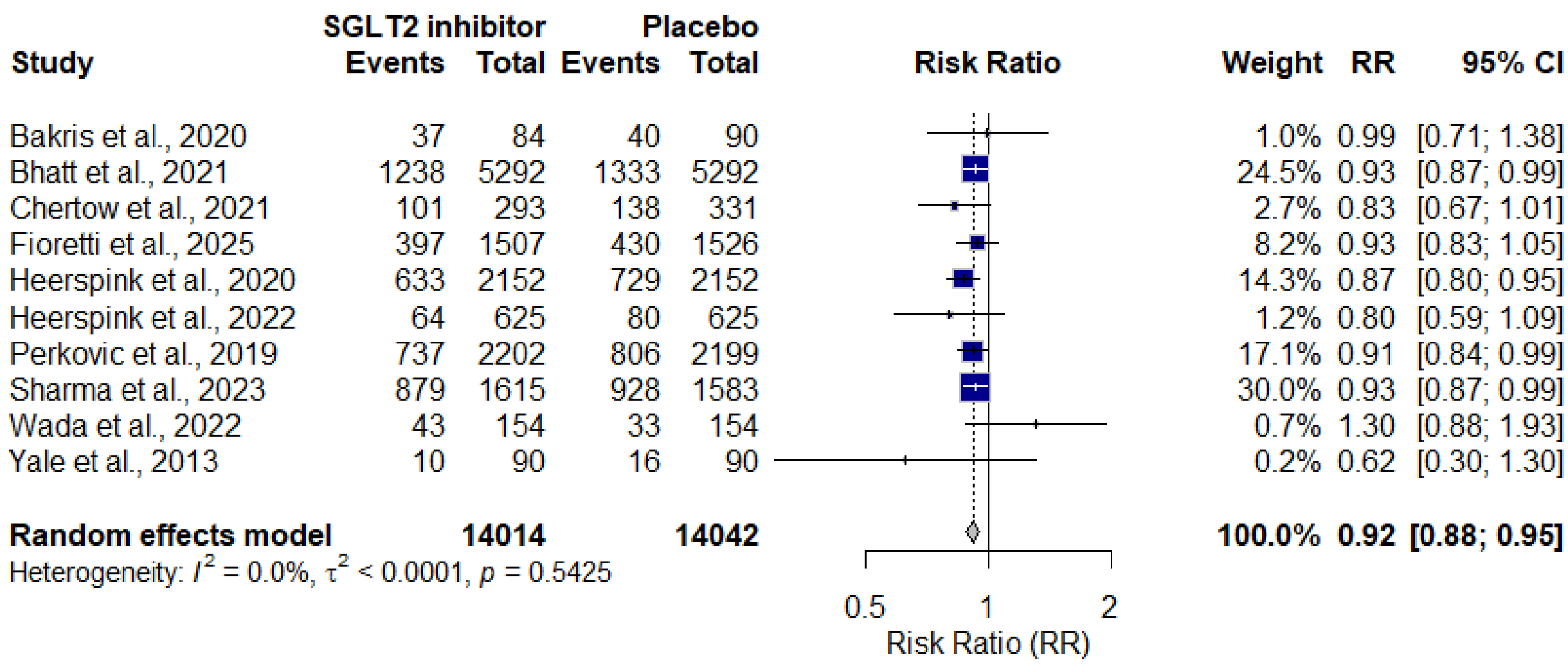

Supplementary Figure S3. Risk ratios for overall incidence of serious adverse events among patients treated with an SGLT2 inhibitor vs. placebo

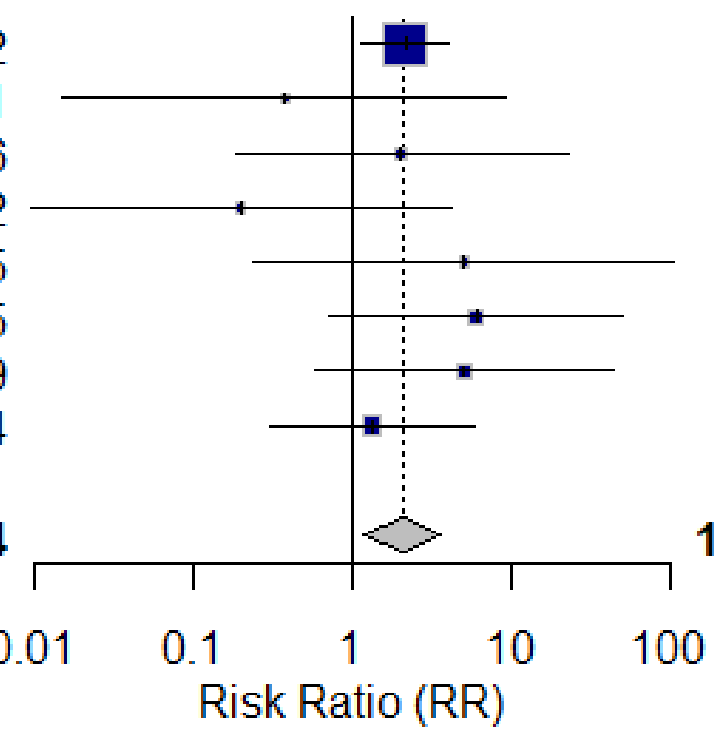

Study	SGLT2 inhibitor Events	SGLT2 inhibitor Total	Placebo Events	Placebo Total	Weight	RR	95% CI
Bhatt et al., 2021	30	5292	14	5292	64.3%	2.14	[1.14; 4.04]
Chertow et al., 2021	0	293	1	331	2.5%	0.38	[0.02; 9.21]
Fioretti et al., 2025	2	1507	1	1526	4.5%	2.03	[0.18; 22.31]
Heerspink et al., 2020	0	2152	2	2152	2.8%	0.20	[0.01; 4.16]
Heerspink et al., 2022	2	625	0	625	2.8%	5.00	[0.24; 103.94]
Herrington et al., 2023	6	3304	1	3305	5.8%	6.00	[0.72; 49.82]
Perkovic et al., 2019	5	2202	1	2199	5.6%	4.99	[0.58; 42.70]
Wada et al., 2022	4	154	3	154	11.8%	1.33	[0.30; 5.86]
**Random effects model**		**15529**		**15584**	**100.0%**	**2.06**	**[1.19; 3.58]**

Heterogeneity: $I^2 = 0.0\%$, $\tau^2 < 0.0001$, $p = 0.5797$

Supplementary Figure S4. Risk ratios for overall incidence of diabetic ketoacidosis (DKA) among patients treated with an SGLT2 inhibitor vs. placebo

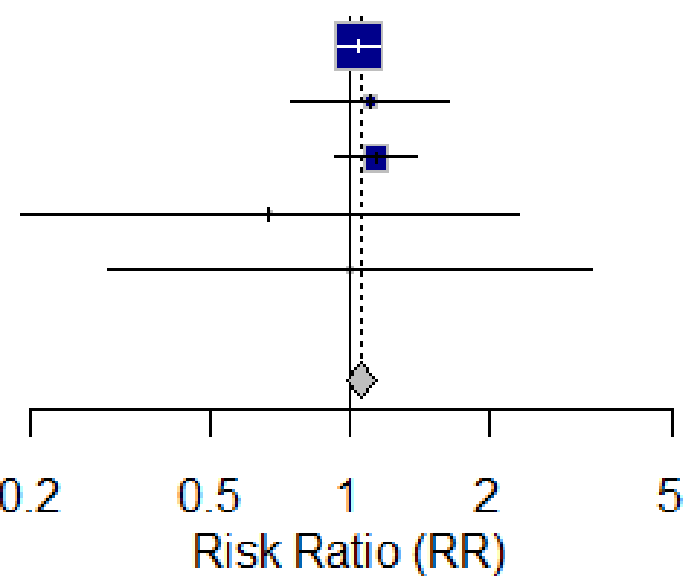

Study	SGLT2 inhibitor Events	SGLT2 inhibitor Total	Placebo Events	Placebo Total	Weight	RR	95% CI
Bhatt et al., 2021	610	5292	585	5292	73.3%	1.04	[0.94; 1.16]
Chertow et al., 2021	43	293	44	331	5.5%	1.10	[0.75; 1.63]
Sharma et al., 2023	178	1615	154	1583	20.1%	1.13	[0.92; 1.39]
Wada et al., 2022	4	154	6	154	0.5%	0.67	[0.19; 2.32]
Yale et al., 2013	5	90	5	90	0.6%	1.00	[0.30; 3.34]
**Random effects model**		**7444**		**7450**	**100.0%**	**1.06**	**[0.99; 1.13]**

Heterogeneity: $I^2 = 0.0\%$, $\tau^2 = 0$, $p = 0.8971$

Supplementary Figure S5. Risk ratios for overall incidence of urinary tract infection (UTI) among patients treated with an SGLT2 inhibitor vs. placebo

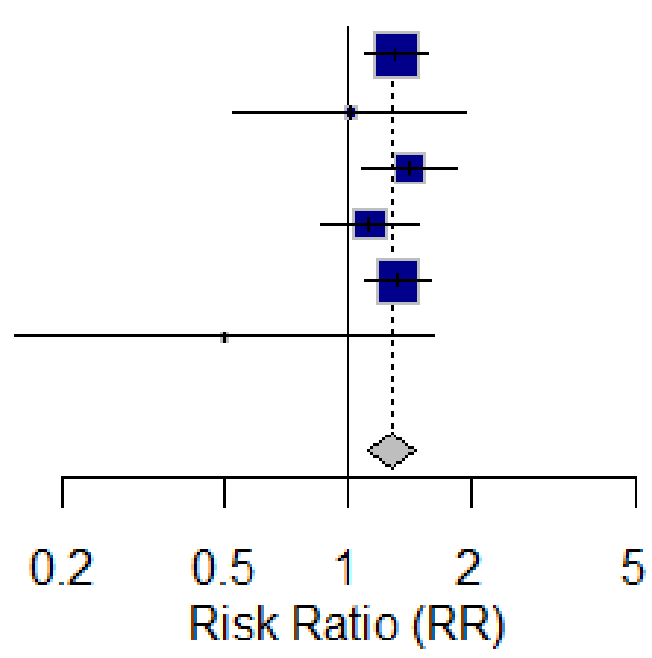

Study	SGLT2 inhibitor Events	SGLT2 inhibitor Total	Placebo Events	Placebo Total	Weight	RR	95% CI
Bhatt et al., 2021	278	5292	213	5292	35.0%	1.31	[1.10; 1.55]
Fioretti et al., 2025	18	1507	18	1526	2.5%	1.01	[0.53; 1.94]
Heerspink et al., 2020	127	2152	90	2152	15.3%	1.41	[1.08; 1.84]
Herrington et al., 2023	113	3304	100	3305	15.2%	1.13	[0.87; 1.47]
Sharma et al., 2023	232	1615	172	1583	31.2%	1.32	[1.10; 1.59]
Wada et al., 2022	4	154	8	154	0.8%	0.50	[0.15; 1.63]
**Random effects model**		**14024**		**14012**	**100.0%**	**1.28**	**[1.13; 1.45]**

Heterogeneity: $I^2 = 0.0\%$, $\tau^2 < 0.0001$, $p = 0.4824$

Supplementary Figure S6. Risk ratios for overall incidence of volume depletion among patients treated with an SGLT2 inhibitor vs. placebo

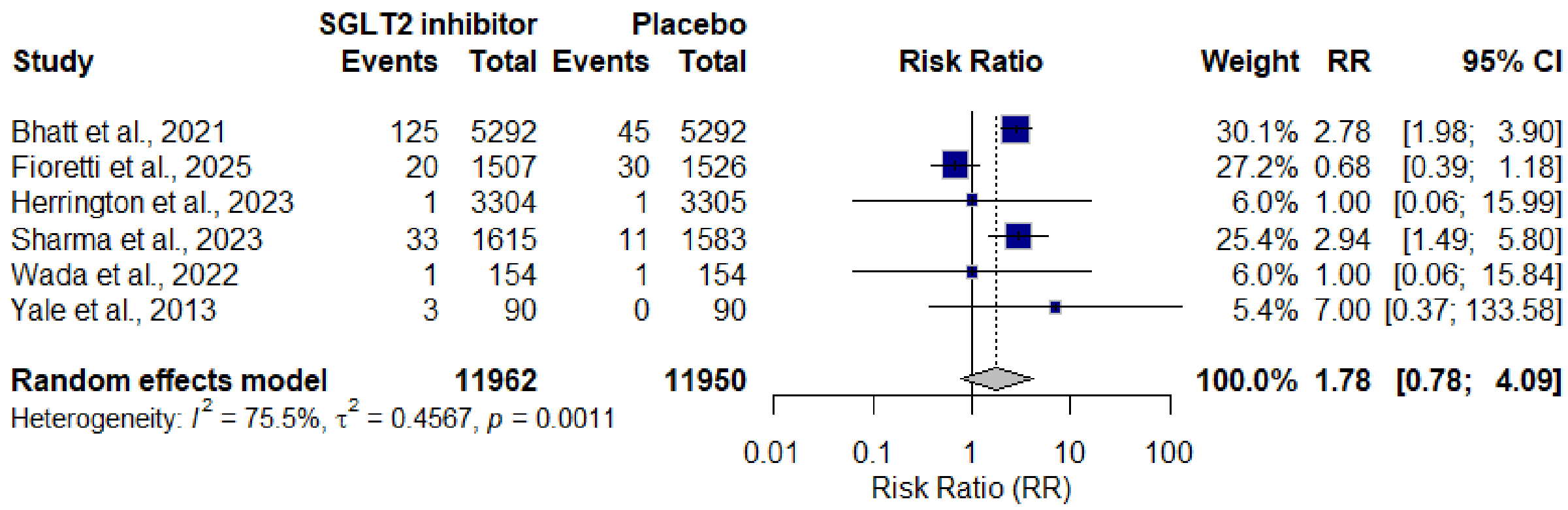

Supplementary Figure S7. Risk ratios for overall incidence of genital infection among patients treated with an SGLT2 inhibitor vs. placebo

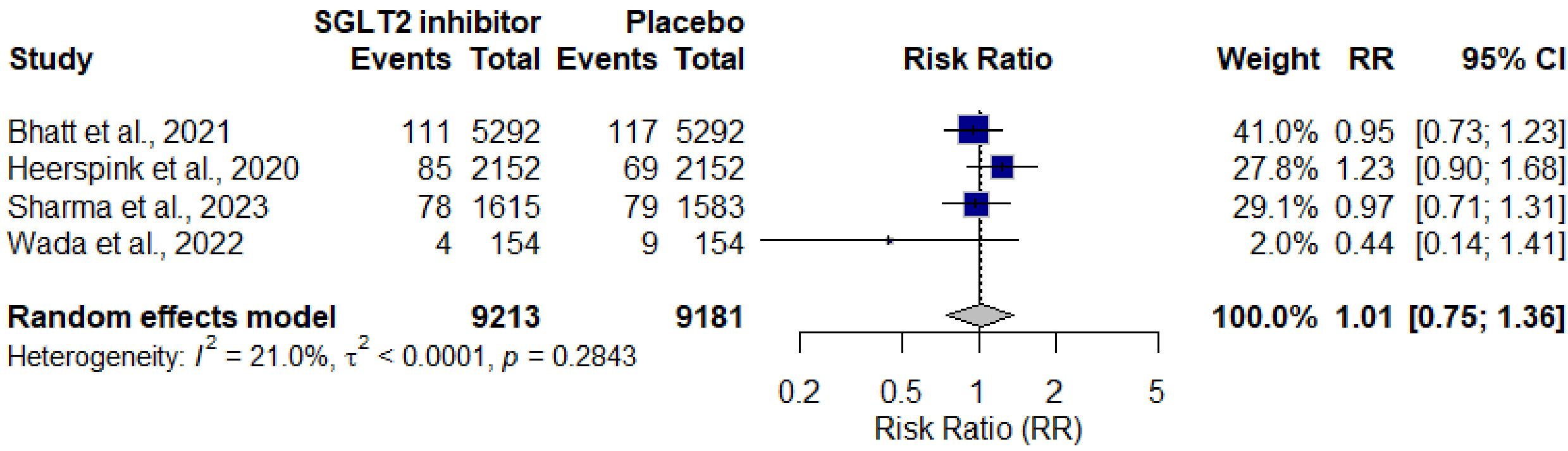

Supplementary Figure S8. Risk ratios for overall incidence of bone fracture among patients treated with an SGLT2 inhibitor vs. placebo

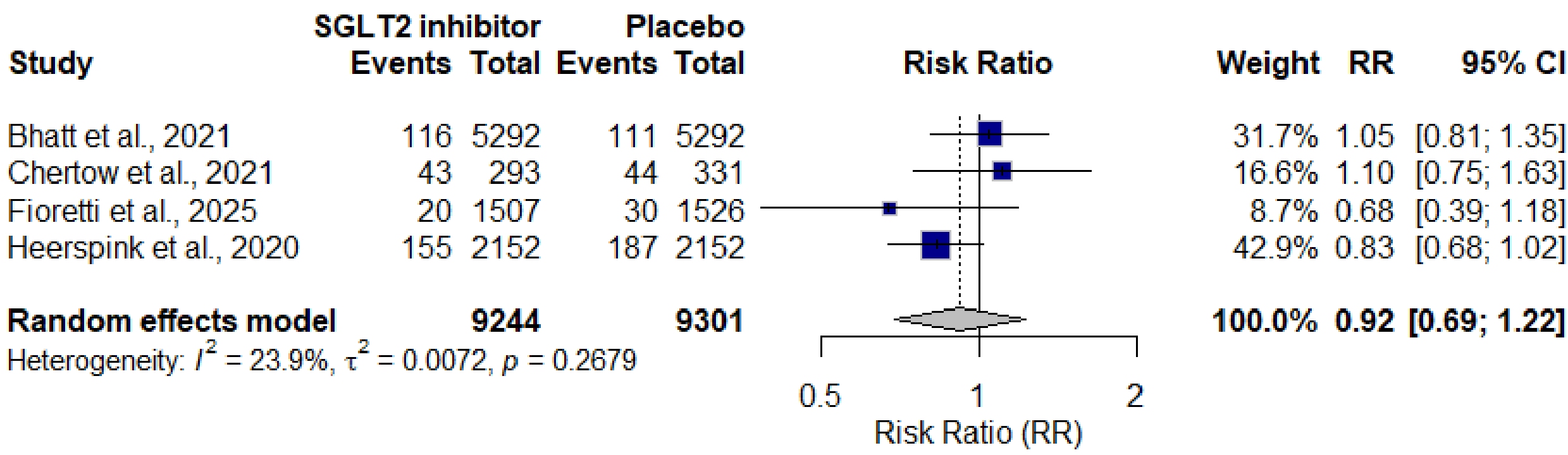

Supplementary Figure S9. Risk ratios for overall incidence of renal-related adverse events among patients treated with an SGLT2 inhibitor vs. placebo

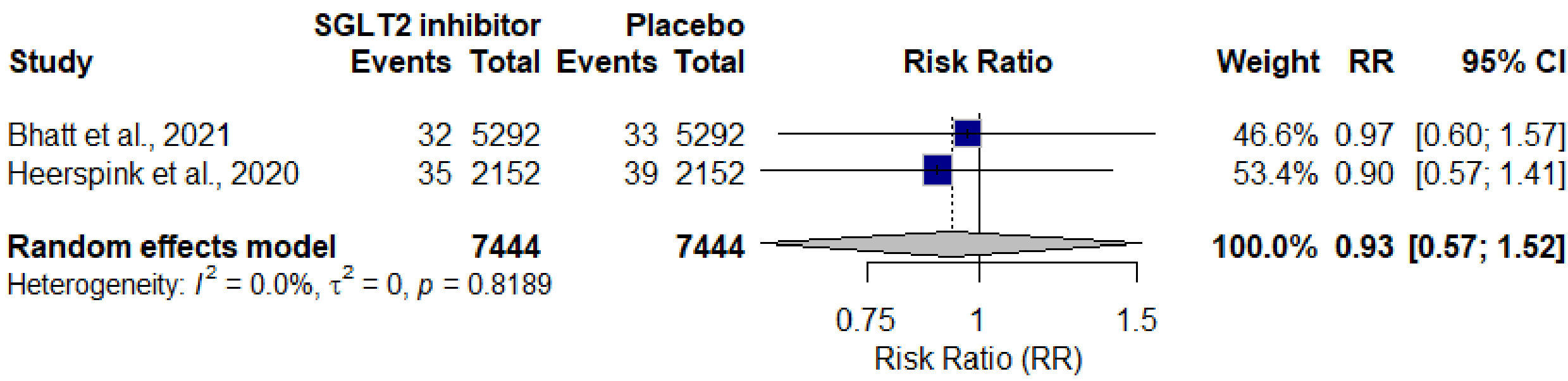

Supplementary Figure S10. Risk ratios for overall incidence of amputation among patients treated with an SGLT2 inhibitor vs. placebo

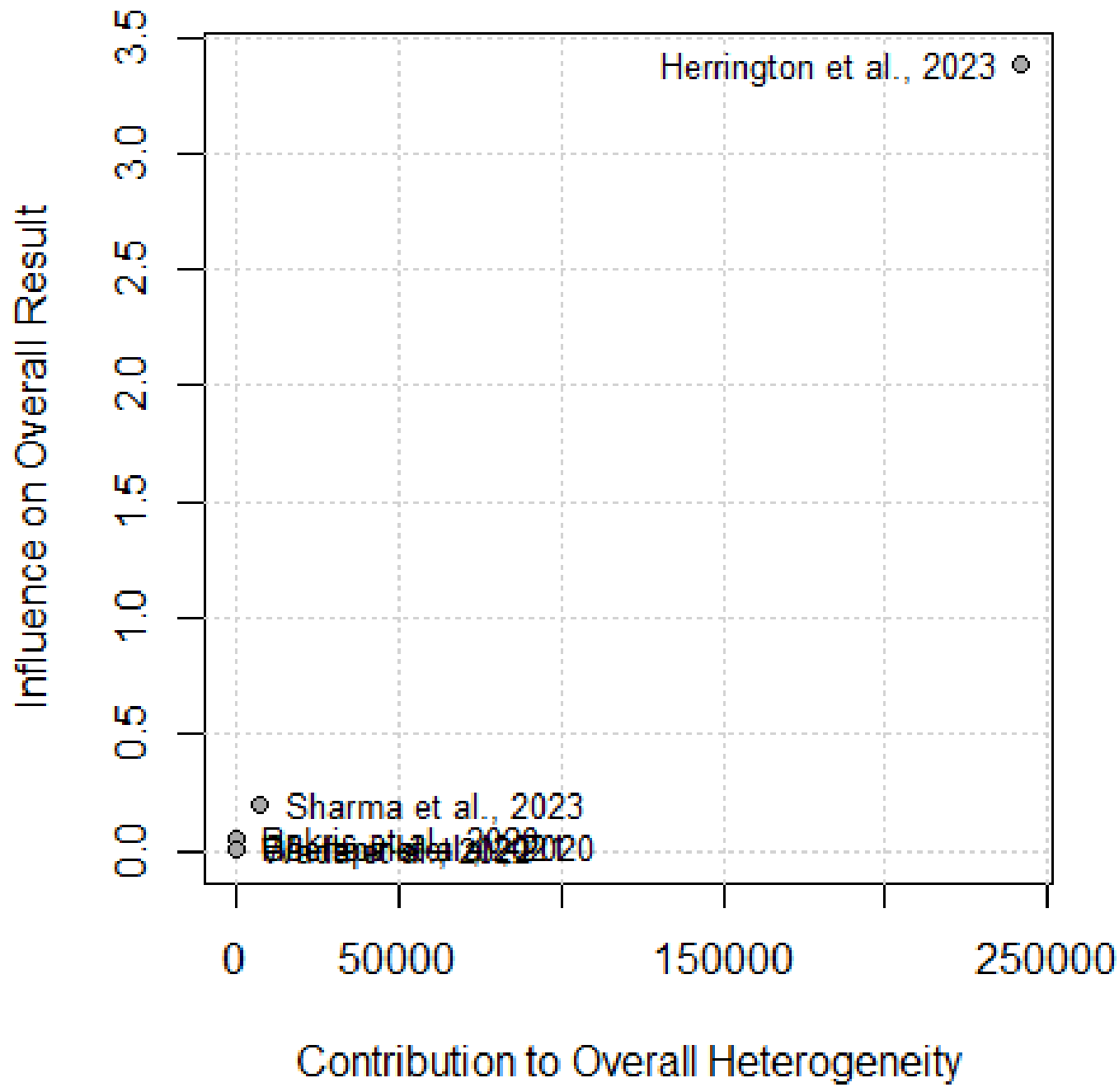

Supplementary Figure S11. Sensitivity analysis. Baujat plot of study-level weighted average effect sizes (outcome = eGFR_total)

Outlier analysis

NULL

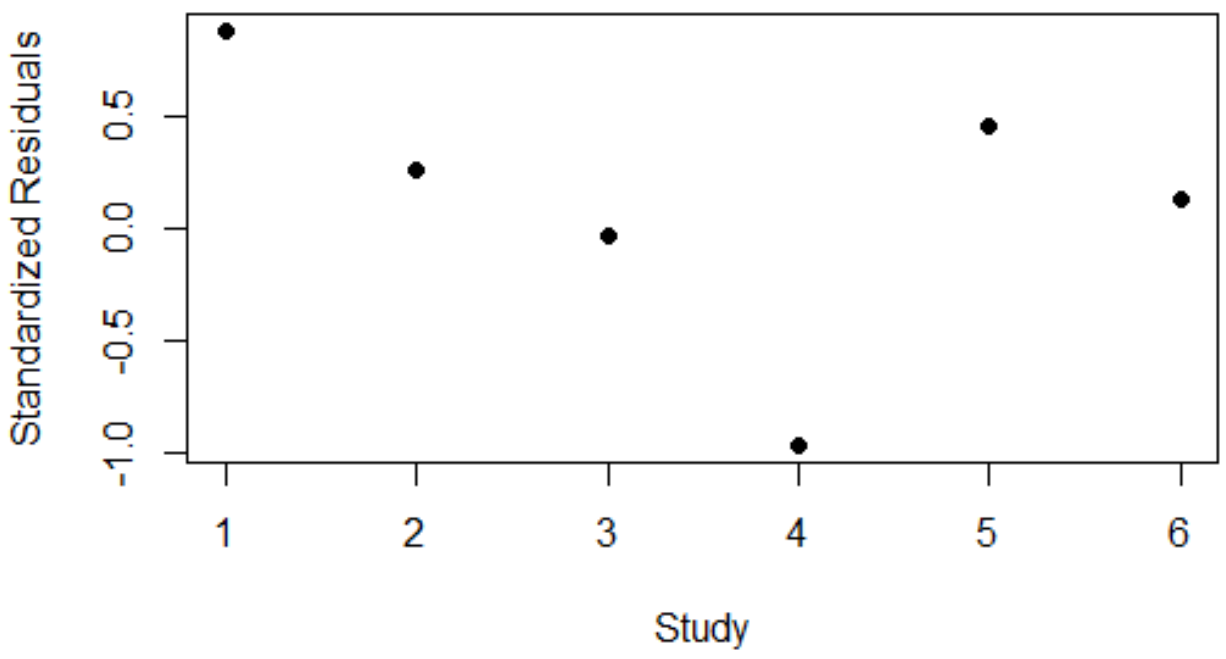

Supplementary Figure S12. Scatterplot for outlier detection (outcome = eGFR_total)

Study 1 = Bakris et al., 2020; Study 2 = Chertow et al., 2021; Study 3 = Heerspink et al., 2020; Study 4 = Herrington et al., 2023; Study 5 = Sharma et al., 2023; Study 6 = Wada et al., 2022

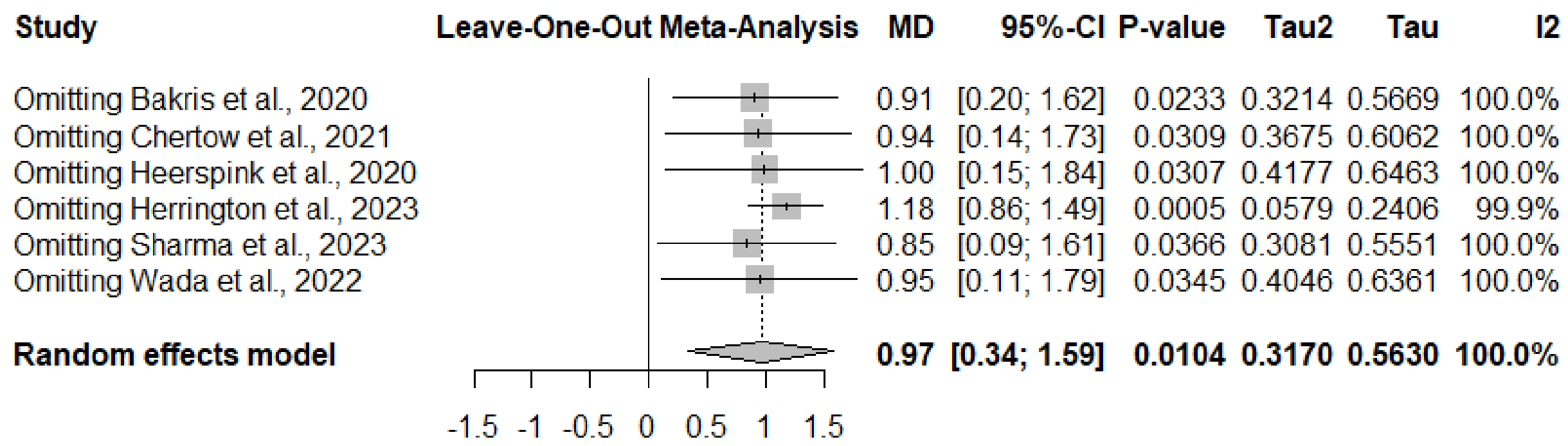

Supplementary Figure S13. Influence Analysis (outcome = eGFR_total)

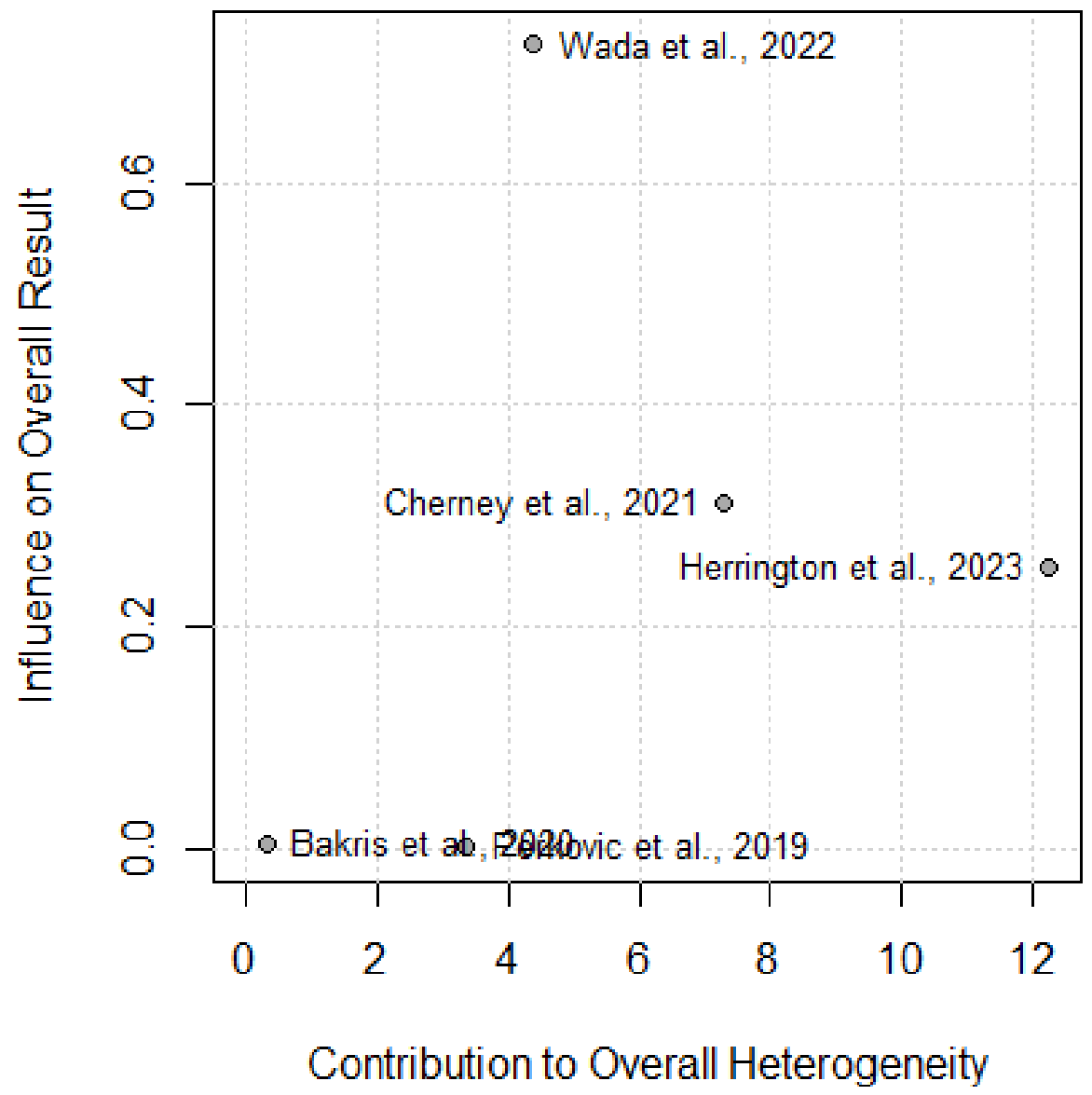

Supplementary Figure S14. Sensitivity analysis. Baujat plot of study-level weighted average effect sizes (outcome = Urine Albumin-Creatinine Ratio (UACR)

Outlier analysis

NULL

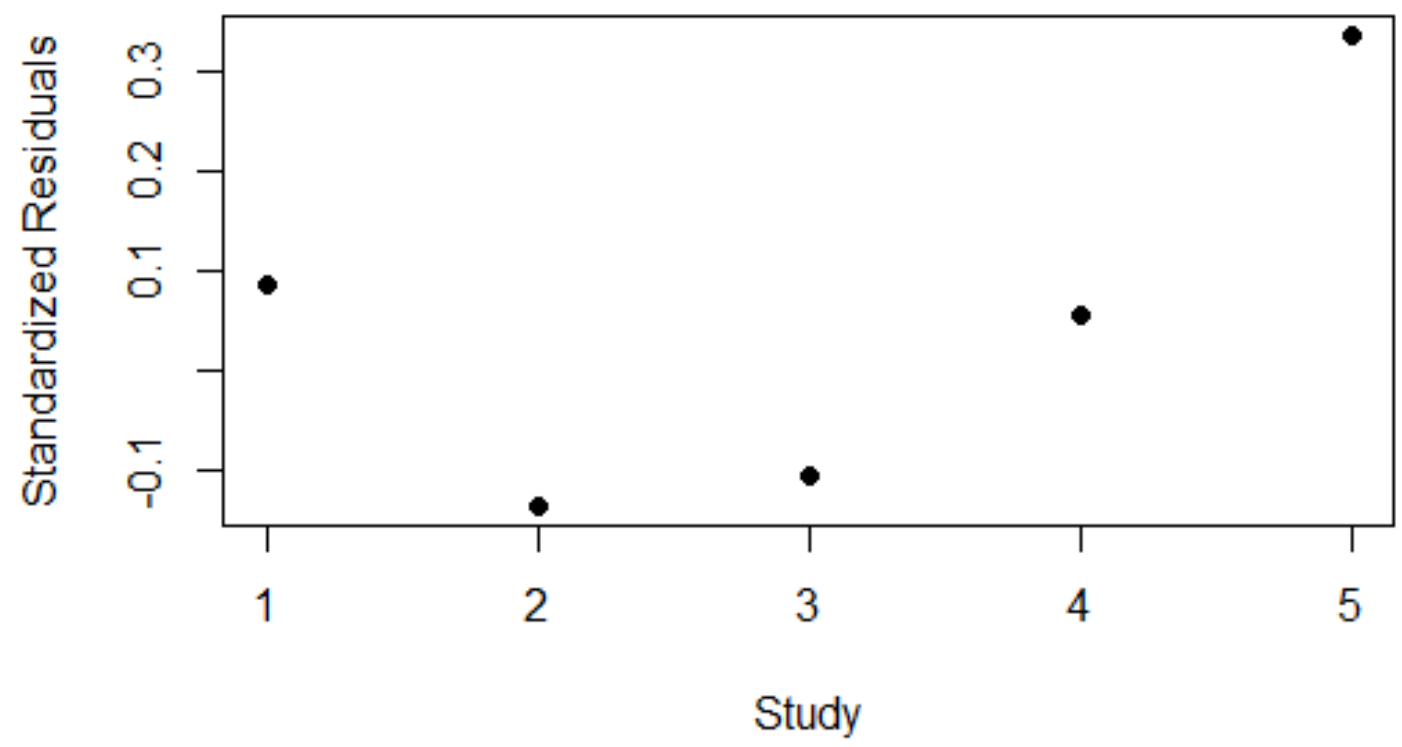

Supplementary Figure S15. Scatterplot for outlier detection (outcome = UACR)

Study 1 = Bakris et al., 2020; Study 2 = Cherney et al., 2021; Study 3 = Herrington et al., 2023; Study 4 = Perkovic et al., 2019; Study 5 = Wada et al., 2022

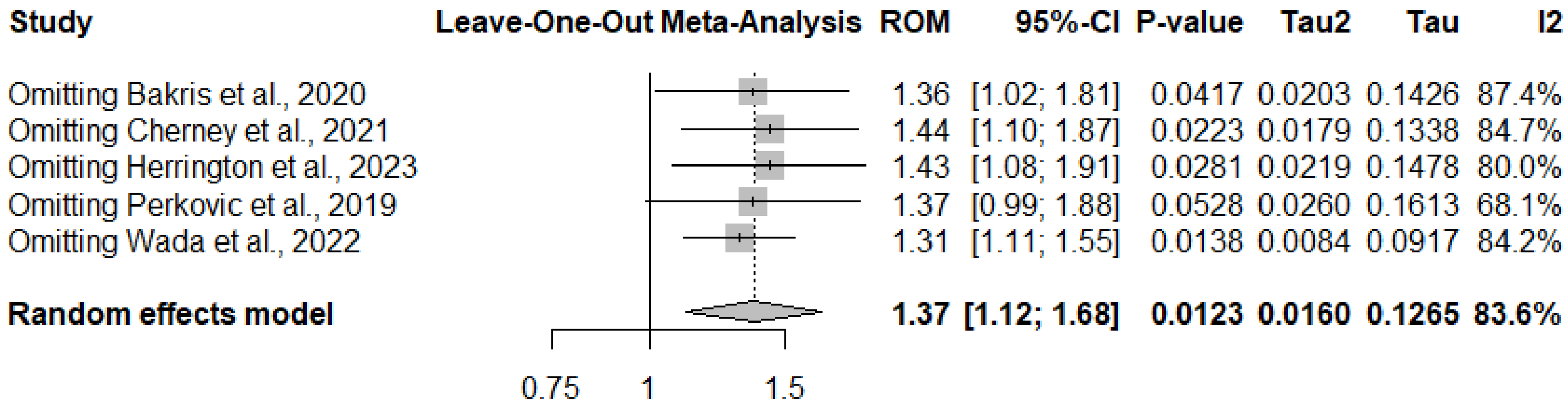

Supplementary Figure S16. Influence Analysis (outcome = UACR)

Supplementary Table S1. Summary of renal, cardiovascular, and safety outcomes in SGLT2 inhibitor trials compared with placebo

Author, primary, year	eGFR Slope, Mean ± SD, mL/min/1.73 m² (annualized total slope)		eGFR Slope, Mean ± SD, mL/min/1.73 m² (annualized chronic slope)		Relative Change in UACR (Ratio of Geometric Means), mg/g		ESKD incidence (Progression to dialysis or transplantation), N		Doubling of Serum Creatinine, N		Hospitalization for heart failure, N		All-cause mortality, N		Cardiovascular mortality, N		Adverse events, N		Serious adverse events, N		DKA, N		UTI, N		Volume depletion, N		Genital infection, N		Bone Fracture, N		Renal related AEs, N		Amputation, N	
	SGLT2i	Placebo	SGLT2i	Placebo	lnROM	SE	SGLT2i	Placebo	SGLT2i	Placebo	SGLT2i	Placebo	SGLT2i	Placebo	SGLT2i	Placebo	SGLT2i	Placebo	SGLT2i	Placebo	SGLT2i	Placebo	SGLT2i	Placebo	SGLT2i	Placebo	SGLT2i	Placebo	SGLT2i	Placebo	SGLT2i	Placebo	SGLT2i	Placebo
Bakris et al., 2020[1]	−0.03 ± 5.72	−1.88 ± 5.92	−1.30 ± 5.41	−3.83 ± 5.60	0.401	0.145	10	14			6	8			8	8	77	81	37	40														
Bhatt et al., 2021[2]							37	52			245	360	246	246	155	170			1238	1333	30	14	610	585	278	213	125	45	111	117	116	111	32	33
Cherney et al., 2021[3]					0.177	0.049	8	4	169	106	231	129																						
Chertow et al., 2021[4]	−2.15 ± 5.48	−3.38 ± 5.64	−1.33 ± 5.48	−3.16 ± 5.64			49	73			18	24	19	31					101	138	0	1	43	44							43	44		
Fioretti et al., 2025[5]											98	144					461	474	397	430	2	1			18	18	20	30			20	30		
Heerspink et al., 2020[6]	−2.86 ± 0.11	−3.79 ± 0.11	−1.67 ± 0.11	−3.59 ± 0.11			109	161	112	210	17	34	101	146	65	80	118	123	633	729	0	2			127	90			85	69	155	187	35	39
Heerspink et al., 2022[7]							13	22	24	33			41	54					64	80	2	0												
Herrington et al., 2023[8]	−2.16 ± 0.08	−2.16 ± 0.08	−1.37 ± 0.08	−1.37 ± 0.08	0.211	0.025	163	217	384	504	131	152	148	167	59	69					6	1			113	100	1	1						
Levin et al., 2020[9]							81	71	81	71			269	194	172	137																		
Perkovic et al., 2019[10]					0.371	0.033	76	100	118	188	89	141	168	201	110	140	1784	1860	737	806	5	1												
Sharma et al., 2023[11]	−0.70 ± 0.15	−2.13 ± 0.15					75	112			189	244	281	427	151	244	1436	1411	879	928			178	154	232	172	33	11	78	79				
Wada et al., 2022[12]	−10.39 ± 0.83	−11.49 ± 0.83			0.654	0.157	4	10			1	3	4	1	3	1	143	140	43	33	4	3	4	6	4	8	1	1	4	9				
Yale et al., 2013[13]													1	1			71	67	10	16			5	5			3	0						

Abbreviations: AEs = Adverse Events; DKA = Diabetic Ketoacidosis; eGFR = Estimated Glomerular Filtration Rate; ESKD = End Stage Kidney Disease; lnROM = Log Ratio of Geometric Means; SD = Standard Deviation; SE = Standard Error; SGLT2i = Sodium Glucose Cotransporter 2 Inhibitors; UACR = Urinary Albumin to Creatinine Ratio; UTI = Urinary Tract Infection

Supplementary Table S2. Risk of bias assessment of randomized controlled studies using revised Cochrane Risk-of-Bias tool (RoB 2)[14]

	Risk of Bias Domains					
**Study**	**D1**	**D2**	**D3**	**D4**	**D5**	**Overall**
**Bakris et al., 2020[1]**	!	!	+	+	+	+
**Bhatt et al., 2021[2]**	!	+	+	+	+	+
**Cherney et al., 2021[3]**	+	!	+	+	+	+
**Chertow et al., 2021[4]**	+	+	+	+	!	+
**Fioretti et al., 2025[5]**	+	!	+	+	+	+
**Heerspink et al., 2020[6]**	+	+	+	+	!	+
**Heerspink et al., 2022[7]**	+	+	!	+	!	+
**Herrington et al., 2023[8]**	+	+	+	+	+	+
**Levin et al., 2020[9]**	!	+	+	+	!	+
**Perkovic et al., 2019[10]**	+	!	!	+	!	!
**Sharma et al., 2023[11]**	+	!	+	+	+	+
**Wada et al., 2022[12]**	!	!	!	+	+	!
**Yale et al., 2013[13]**	+	+	!	+	!	+

+ Low risk

! Some concerns

- High risk

D1 = Randomisation process

D2 = Deviations from the intended interventions

D3 = Missing outcome data

D4 = Measurement of the outcome

D5 = Selection of the reported result

References

1. Bakris G, Oshima M, Mahaffey KW, et al. Effects of Canagliflozin in Patients with Baseline eGFR <30 ml/min per 1.73 m2: Subgroup Analysis of the Randomized CREDENCE Trial. *Clin J Am Soc Nephrol*. 2020;15(12):1705-1714. doi:10.2215/CJN.10140620

2. Bhatt DL, Szarek M, Pitt B, et al. Sotagliflozin in Patients with Diabetes and Chronic Kidney Disease. *N Engl J Med*. 2021;384(2):129-139. doi:10.1056/NEJMoa2030186

3. Cherney DZI, Charbonnel B, Cosentino F, et al. Effects of ertugliflozin on kidney composite outcomes, renal function and albuminuria in patients with type 2 diabetes mellitus: an analysis from the randomised VERTIS CV trial. *Diabetologia*. 2021;64(6):1256-1267. doi:10.1007/s00125-021-05407-5

4. Chertow GM, Vart P, Jongs N, et al. Effects of Dapagliflozin in Stage 4 Chronic Kidney Disease. *J Am Soc Nephrol*. 2021;32(9):2352-2361. doi:10.1681/ASN.2021020167

5. Fioretti F, Butler J, Udell JA, et al. Empagliflozin After Myocardial Infarction with or without diabetes and chronic kidney disease: Insights from EMPACT-MI. *ESC Heart Fail*. 2025;12(6):3940-3952. doi:10.1002/ehf2.15393

6. Heerspink HJL, Stefánsson BV, Correa-Rotter R, et al. Dapagliflozin in Patients with Chronic Kidney Disease. *N Engl J Med*. 2020;383(15):1436-1446. doi:10.1056/NEJMoa2024816

7. Heerspink HJL, Furtado RHM, Berwanger O, et al. Dapagliflozin and Kidney Outcomes in Hospitalized Patients with COVID-19 Infection: An Analysis of the DARE-19 Randomized Controlled Trial. *Clin J Am Soc Nephrol*. 2022;17(5):643-654. doi:10.2215/CJN.14231021

8. The EMPA-KIDNEY Collaborative Group. Empagliflozin in Patients with Chronic Kidney Disease. *N Engl J Med*. 2023;388(2):117-127. doi:10.1056/NEJMoa2204233

9. Levin A, Perkovic V, Wheeler DC, et al. Empagliflozin and Cardiovascular and Kidney Outcomes across KDIGO Risk Categories: Post Hoc Analysis of a Randomized, Double-Blind, Placebo-Controlled, Multinational Trial. *Clin J Am Soc Nephrol*. 2020;15(10):1433-1444. doi:10.2215/CJN.14901219

10. Perkovic V, Jardine MJ, Neal B, et al. Canagliflozin and Renal Outcomes in Type 2 Diabetes and Nephropathy. *N Engl J Med*. 2019;380(24):2295-2306. doi:10.1056/NEJMoa1811744

11. Sharma A, Ferreira JP, Zannad F, et al. Cardiac and Kidney Benefits of Empagliflozin in Heart Failure Across the Spectrum of Kidney Function: Insights from the EMPEROR-Preserved Trial. *Eur J Heart Fail*. 2023;25(8):1337-1348. doi:10.1002/ejhf.2857

12. Wada T, Mori-Anai K, Takahashi A, et al. Effect of canagliflozin on the decline of estimated glomerular filtration rate in chronic kidney disease patients with type 2 diabetes mellitus: A multicenter, randomized, double-blind, placebo-controlled, parallel-group, phase III study in Japan. *J Diabetes Investig*. 2022;13(12):1981-1989. doi:10.1111/jdi.13888

13. Yale J -F., Bakris G, Cariou B, et al. Efficacy and safety of canagliflozin in subjects with type 2 diabetes and chronic kidney disease. *Diabetes Obes Metab*. 2013;15(5):463-473. doi:10.1111/dom.12090

14. Sterne JAC, Savović J, Page MJ, et al. RoB 2: a revised tool for assessing risk of bias in randomised trials. *BMJ*. Published online August 28, 2019:l4898. doi:10.1136/bmj.l4898
